# To Cooperate or Not to Cooperate: Why Behavioural Mechanisms Matter

**DOI:** 10.1371/journal.pcbi.1004886

**Published:** 2016-05-05

**Authors:** Arthur Bernard, Jean-Baptiste André, Nicolas Bredeche

**Affiliations:** 1 Sorbonne Universités, UPMC Univ Paris 06, CNRS, Institute of Intelligent Systems and Robotics (ISIR), Paris, France; 2 Institut des Sciences de l’Evolution, Université de Montpellier, CNRS, IRD, EPHE, CC065, Pl. E. Bataillon, Montpellier, France; Michigan State University, UNITED STATES

## Abstract

Mutualistic cooperation often requires multiple individuals to behave in a coordinated fashion. Hence, while the evolutionary stability of mutualistic cooperation poses no particular theoretical difficulty, its evolutionary emergence faces a chicken and egg problem: an individual cannot benefit from cooperating unless other individuals already do so. Here, we use evolutionary robotic simulations to study the consequences of this problem for the evolution of cooperation. In contrast with standard game-theoretic results, we find that the transition from solitary to cooperative strategies is very unlikely, whether interacting individuals are genetically related (cooperation evolves in 20% of all simulations) or unrelated (only 3% of all simulations). We also observe that successful cooperation between individuals requires the evolution of a specific and rather complex behaviour. This behavioural complexity creates a large fitness valley between solitary and cooperative strategies, making the evolutionary transition difficult. These results reveal the need for research on biological mechanisms which may facilitate this transition.

## Author Summary

Mutualistic behaviours wherein several individuals act together for a common benefit, such as a collective hunt, are often deemed of minor interest by theoreticians in evolutionary biology. These behaviours benefit all the individuals involved, and therefore, so the argument goes, their evolution is straightforward. However, mutualistic behaviours do pose a specific kind of evolutionary problem: they require the coordination of several partners. Indeed, a single individual expressing a preference for cooperation cannot benefit if others wish to remain solitary. Here we use simulations in evolutionary robotics to study the consequences of this problem. We show that it constitutes a far more serious obstacle for the evolution of cooperation than was previously thought on the basis of game theoretical analyses. We find that the transition from solitary to cooperative strategies is very unlikely, and we also observe that successful cooperation requires the evolution of a specific and rather complex behaviour, necessary for individuals to coordinate with each other. This reveals the critical role of the practical mechanics of behaviour in evolution.

## Introduction

It is well known that, in the absence of genetic relatedness, altruistic behaviours in which individuals pay a fitness cost for the benefit of others cannot evolve by natural selection [1, 2]. However, it is often assumed that mutualistic behaviours, wherein individuals collectively gain a common benefit [28, 29], do not pose such a problem, and are therefore of limited interest to evolutionists: they simply evolve because they benefit the individuals who express them.

However, mutualistic behaviours do often pose a different kind of evolutionary problem than altruism: they require coordination [3, 5–28]. Many collective traits are only mutually beneficial if several individuals express them together in a coordinated fashion. That is, it would not be beneficial for a single individual to express the cooperative trait if others did not express it as well. Consequently, whereas altruistic behaviours pose a problem of *stability*, which can only be solved by genetic relatedness, many forms of mutualistic behaviours pose a problem of *evolution*. These collective strategies are stable equilibria but their evolution is complex.

This problem has been formalized in game theory as the stag hunt game [6]. In the stag hunt, two hunters are confronted with the choice of either hunting a hare alone for a small but guaranteed benefit, or coordinating to hunt a stag cooperatively for a bigger reward, with the risk of not being rewarded at all if they hunt the stag alone. There are two evolutionarily stable Nash equilibria in this game: (1) simultaneous defection (i.e. both players hunt hares), which is risk-dominant as it maximizes the minimum payoff an individual can expect, and (2) simultaneous cooperation (i.e. both players hunt stags), which is payoff-dominant as it maximizes the total payoff at equilibrium. One of the aims of evolutionary analyses of the stag hunt is to characterize the mechanisms that facilitate the transition from the solitary equilibrium to the cooperative equilibrium. The difficulty is that cooperation can only be favoured by selection when a sufficient proportion of individuals in the population also cooperate. The transition from a population with a majority of solitary individuals to one with a majority of social individuals requires the rise of cooperation above an invasion threshold, which must occur for non-selective reasons.

In game-theoretic analyses, the hunting strategy of individuals is generally assumed to be encoded by a single genetic locus with two alleles: solitary or social [6]. In this case, random mutations and/or demographic stochasticity can lead to the appearance of a subpopulation of mutants playing the social strategy which is sufficient to overcome the invasion threshold. Moreover, Skyrms [6] showed that this cooperation can be further facilitated in a spatially structured population in which individuals tend to interact more with genetically related partners.

However, this approach makes a very strong assumption about the underlying mechanistic nature of behaviour: that a single mutation is sufficient to transform an individual playing a solitary strategy into an individual playing a perfectly efficient social strategy. In reality, hunting socially implies several novel behavioural abilities. In particular, it implies the ability to coordinate with others in order to focus on the same prey, which is unlikely to occur with only a single random mutation. In this paper, we postulate that critical aspects of coordinated cooperation have been neglected by game-theoretic analyses and investigate the mechanistic constraints which interfere with the evolution of coordination in a more realistic setting where the mapping between genotype and phenotype is not limited to a strict binary encoding.

Evolutionary robotics is a useful methodology for the simulation and study of this more realistic conception of behaviour and its genetic underpinnings [7, 8]. This approach allows to simulate the evolution of complex genotypes and observe the resulting behaviours in robotic agents. Such simulations also make it possible to investigate the complex mechanistic constraints at play in the translation from genotype to phenotype [24]. A considerable body of work has already been dedicated to modeling social evolution with robotic approaches [14]. These studies have been interested in a large diversity of issues: the evolution of swarms [11], the mechanics of division of labour in social insects [15, 16] or the evolution of communication [10, 17–19]. The evolution of cooperation in particular has been addressed in numerous papers. In the vast majority of this literature, however, social partners are genetically related [20], whether motivated by design [21, 22] or to study the evolution of altruism [9, 23]. Few articles, in comparison, have been interested in the evolution of mutualistic cooperation between genetically unrelated individuals [19]. Moreover the specific problem posed by the stag hunt game, where cooperation is not the only evolutionarily stable strategy and a non-collective solution acts as a stable attractor, has never been studied in evolutionary robotics.

In this paper, we use an experimental model where simulated robotic agents interact in a situation equivalent to the stag hunt and compare the results of our model to those of standard game-theoretic analyses. Our results shed new light on the influence of mechanistic constraints in the evolution of coordinated actions. We then use this model to explore realistic mechanisms that could drive the transition to collective behaviours.

## Materials and Methods

### Experimental setup

**Fig 1:**
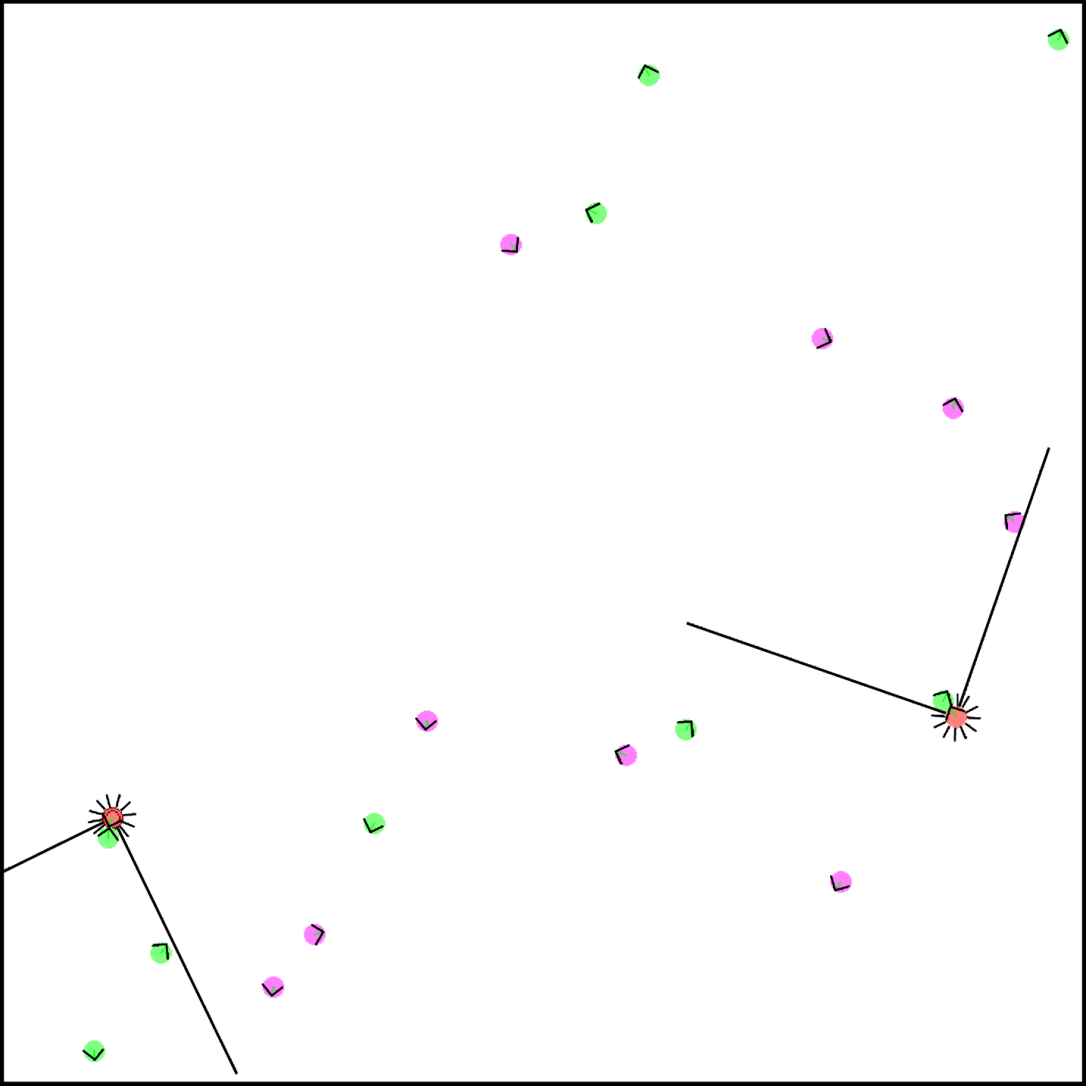
Screenshot of a robotic simulation. The red dots represent the two hunters, the green dots the hares, and the pink dots the stags. The black lines around the agents’ body represent the proximity sensors and the black cones on front the cameras described in the text. Hunters are allowed to move throughout the environment. Hares and stags remain at their starting positions.

We consider an environment with two hunters and several prey, both hares and stags. Hunters can choose to hunt either of these prey, earning different food rewards depending on whether they hunt alone or cooperate (see Fig 1).

Food rewards for killing a prey are shown in Table 1. A hare yields a reward of 50, regardless of whether it is hunted in a solitary or cooperative fashion. A stag yields a reward of 500 for each hunter only if it is hunted cooperatively. If a stag is killed by a single hunter, it is still removed from the arena but is considered a failed hunt and rewards nothing. None of the rewards are split between cooperators.

**Table 1:** Food rewards for hunting different prey.

Prey		Food Reward
Hare	*alone*	50
	*coop.*	50
Stag	*alone*	0
	*coop.*	500

The reward depends on whether these prey were hunted alone or cooperatively. There is no reward for stags hunted alone in this case.

Simulated robotic agents are evaluated in an 800 by 800 unit square arena, which has four solid walls and is devoid of any obstacles aside from other agents. Each circular-shaped agent, with a diameter of 14 units, is equipped with two independent wheels and a collection of sensors. Hunters can use the information provided by 12 proximity sensors and a front camera. Proximity sensors have a range of approximately twice the diameter of the agent’s body, and provide the agent with the proximity of the nearest obstacle. They are evenly distributed around the agent’s body. The front camera consists of 12 rays with infinite range spread out in a 90 degree cone in front of the body. Each ray in the camera provides two different pieces of information about the first target it intersects with: the type of target (hunter, hare, or stag) and its proximity. This robot model facilitates the evolution of basic walls avoidance and agents recognition behaviours, which we consider not to be of interest here. Hence we separate obstacles recognition (by the proximity sensors) from agents’ recognition (by the camera).

Only the hunters are capable of movement; prey remain at their initial positions. (Complementary experiments with moving prey capable of avoidance behaviours did not produce significantly different results; not shown.) A prey is caught if any hunter remains close enough during a fixed amount of time steps (800 steps, in a simulation lasting 20.000 time steps). Cooperative hunting is defined as a prey with two hunters in catching distance at the time of its capture. Therefore, cooperation happens even if only one of the two hunters is in catching distance of the prey for most of the time, as long as the two hunters are there in the final step. The prey is then immediately replaced at a random position in the arena, thus keeping a fixed number of agents and prey during the whole simulation.

### Neural network for agent control

**Fig 2:**
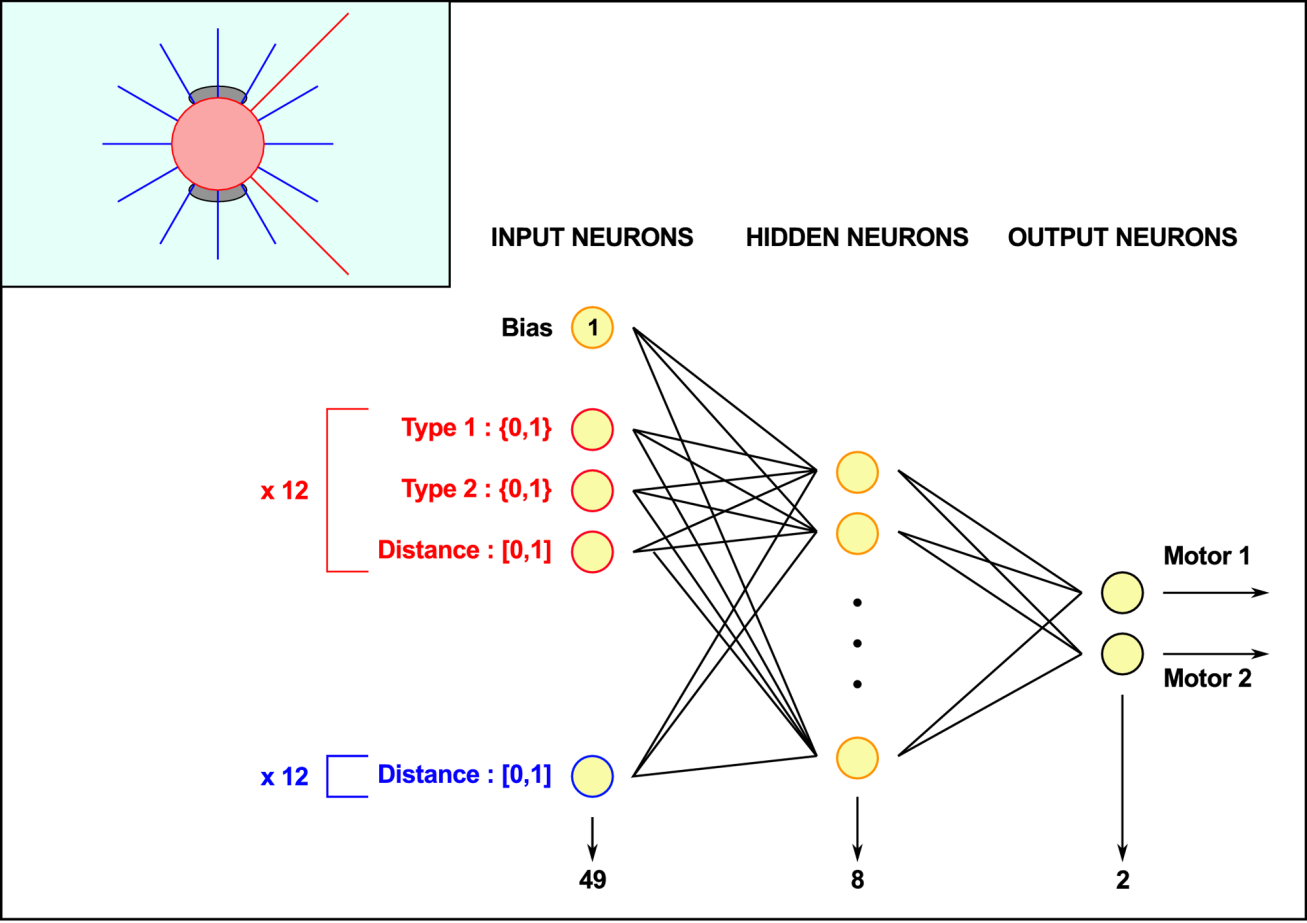
Diagram of the simulated robotic agent used in the simulation (inset) and its neural network controller. The blue lines represent the 12 proximity sensors and the red lines represent the front camera. Inputs “Type 1” and “Type 2” are two boolean values used to represent the type of the agent (encoded with two bits) recognized by the camera ray.

The hunters’ behaviour is computed by an artificial neural network which maps sensory inputs to motor outputs. The neural network is a fully connected multi-layer perceptron with a single hidden layer of 8 neurons. The inputs of this network are the perceptions of the agent, with 12 neurons for the proximity sensors and 48 for the camera (4 for each of the 12 rays) plus a bias neuron (whose value is always 1), for a total of 61 input neurons. The two outputs of the network control the speed of each of the agent’s wheels and the mapping function between inputs and outputs is a sigmoid function (see Fig 2). Changing the number of hidden neurons did not yield significantly different results (not shown).

### Simulating artificial evolution

To simulate evolution, we use an evolutionary algorithm to evolve the genome of the hunters. This genome is comprised of a collection of 410 real values in the range [0, 1], one for each of the neural network’s weights, and is initially randomized for each individual in the population. In order to obtain its fitness, each individual is successively paired five times with a partner randomly chosen each time (except itself) in the arena presented in the Experimental Setup subsection, for an evaluation round of 20.000 time steps. The payoff of the evaluated individual at the end of a round is given by the total amount of food it has managed to obtain by killing prey in this round. As this quantity depends heavily on the initial conditions (random initial positions of the prey), five simulations are performed for each pair of individuals. The individual’s fitness is then obtained by computing the sum of payoffs averaged over the total number of simulations for the individual. In this case the number of simulations is 25, with 5 partners and 5 simulations with each partner.

Experiments were conducted using a Wright-Fisher model [12] with constant population size (20 individuals), which is commonly known as a fitness-proportionate selection method in evolutionary robotics [13]. Using this model, the population of the next generation is formed by a random sampling of offspring from the previous generation, with the probability of sampling a particular parent proportional to the parent’s fitness. Each offspring is simply a mutated clone of its parent; recombination is not included in our simulation. Consequently, new genotypes appear only through mutation. These mutations are performed using a Gaussian function, with a standard deviation of 2 × 10^−1^ and a mutation probability of 5 × 10^−3^. Each experiment lasted 3000 generations. All simulation parameters are summarised in Table 2.

**Table 2:** Simulation parameters.

Parameter		Value
**Evolutionary Algorithm**		
	Selection method	Fitness-proportionate
	Population size	20
	Gene mutation probability	5 × 10^−3^
	Mutation operator	Gaussian N(0,0.01)
	Number of partners	5
	Number of simulations per pair	5
**Artificial Neural Network**		
	Input neurons	61
	Hidden neurons	8
	Output neurons	2

## Results

### Starting with a population of hare hunters

**Fig 3:**
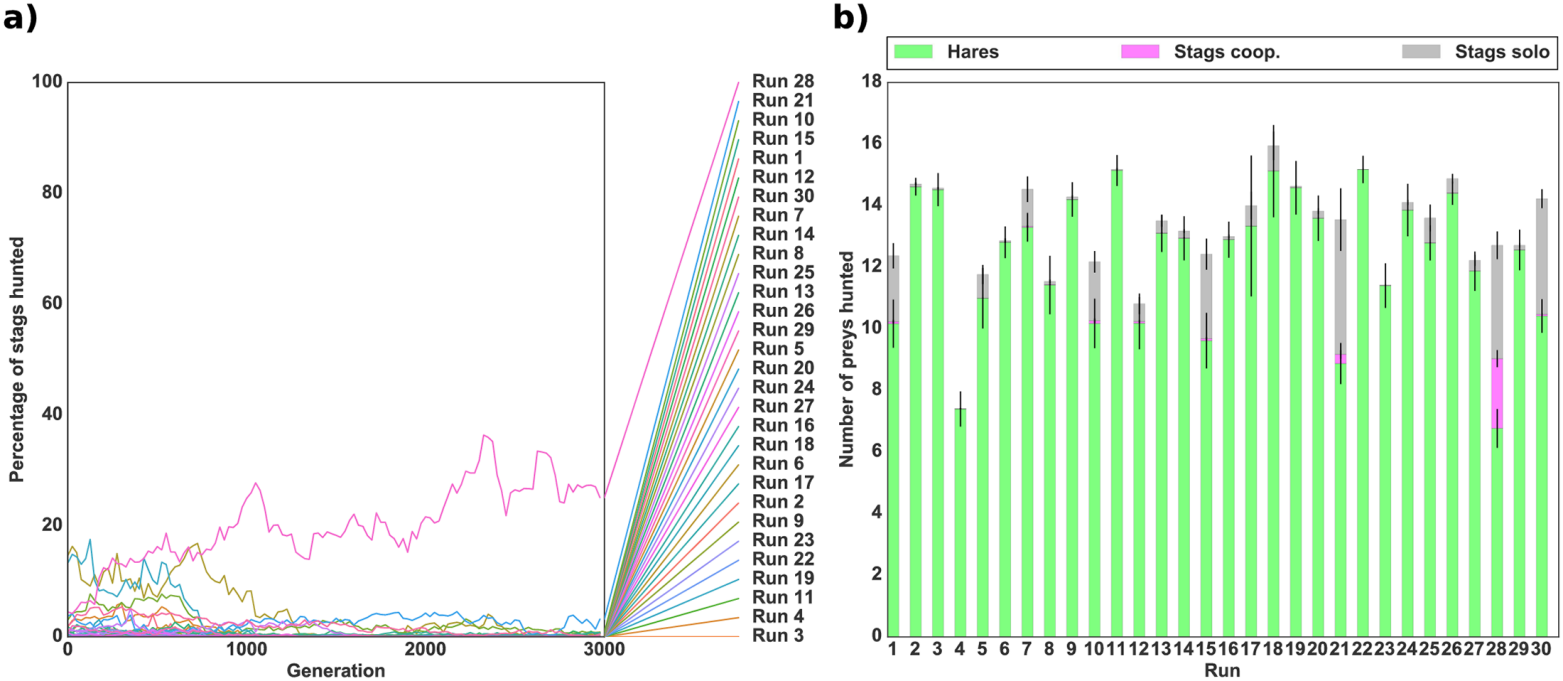
Evolution of cooperation in a robotic simulation with an initial hare-hunting strategy. *(a)* Evolution of the mean percentage of stags hunted successfully (i.e. cooperatively) with respect to the total number of prey hunted. *(b)* Mean number of prey hunted during the last generation of evolution for each independent run. The bottom green bar represents the number of hares hunted, the middle pink bar the number of stags hunted successfully (cooperatively), and the top grey bar the number of failed hunts (stags hunted alone). The standard deviation for each quantity is shown by black lines. The population for each of the 30 independent runs was previously evolved in an environment with only hares. Rewards were 50 for a hare, 0 for a stag hunted alone, and 500 for a stag hunted cooperatively as presented in Table 1. The number of prey (18) was kept constant throughout the simulation by replacing killed prey by a prey of the same type.

In order to explore the evolutionary transition between the risk-dominant equilibrium (hare hunting) and the payoff-dominant equilibrium (cooperative stag hunting), individuals first evolved in an environment composed solely of hares. This ensured that the populations initially reached the solitary equilibrium. Only then did we add stags and study the dynamics of evolution. Fig 3(a) shows the evolution of the mean percentage of stags hunted successfully (i.e., hunted cooperatively) out of the total number of prey hunted over time for 30 independent runs. Fig 3(b) shows the mean proportion of each type of prey hunted during the last generation of each run. Stag hunting evolved in only one run out of 30 and even in that run accounted for less than 30% of the total number of prey hunted. In the other 29 runs, the individuals hunted only hares as they had previously evolved to do. These simulations demonstrate that the evolution of collective hunting is very unlikely when the population is composed of individuals who are already efficient solitary hunters.

**Fig 4:**
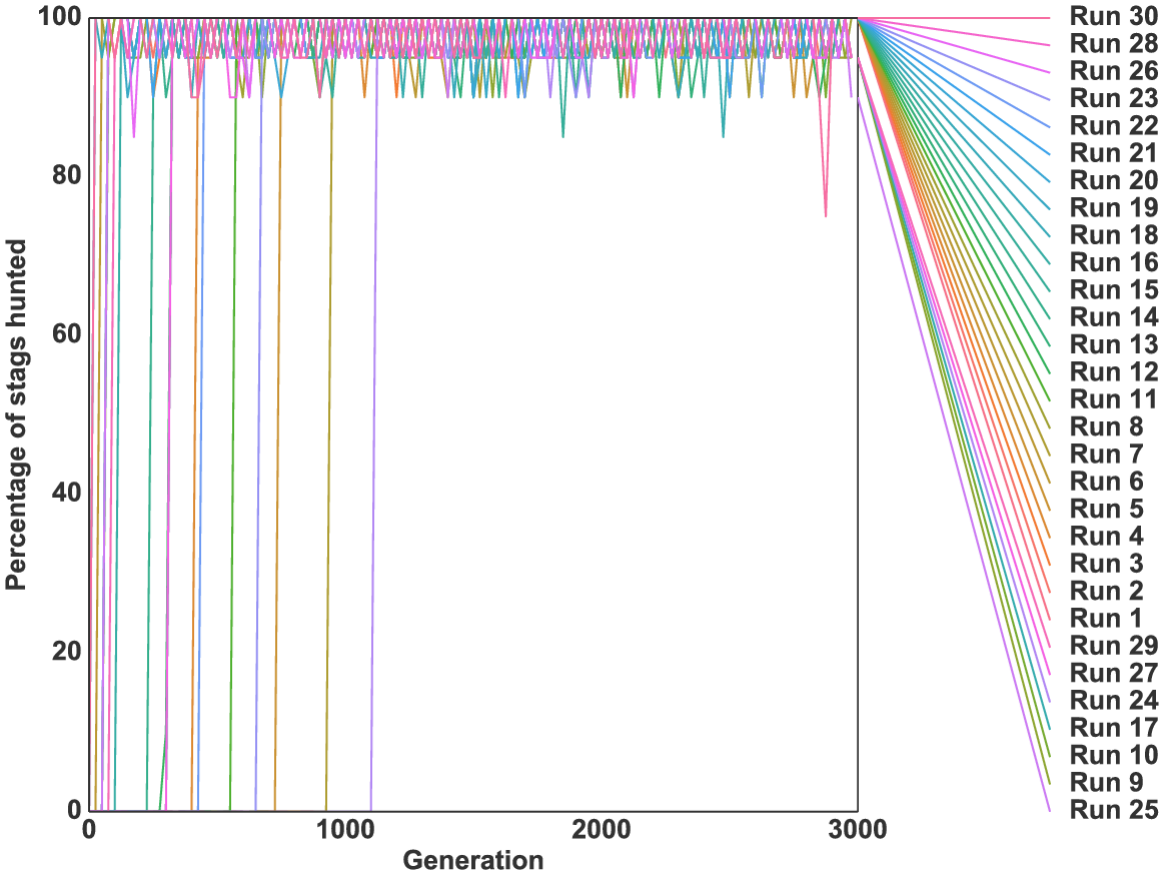
Evolution of cooperation in a game-theoretic simulation with an initial hare-hunting strategy. Evolution of the mean percentage of stags hunted successfully (i.e. cooperatively) with respect to the total number of prey hunted when starting with a population of hare hunters for 30 independent runs. Rewards were 50 for a hare, 0 for a stag hunted alone, and 500 for a stag hunted cooperatively as presented in Table 1.

For comparison we simulated the same scenario using the standard game-theoretic version of the stag hunt, where the expression of the two types of behaviour was encoded by a single binary locus. Each individual in the population initially possessed the allele for hare hunting (Fig 4).

Here the transition to collective hunting occurred in each of the 30 independent runs and this strategy then remained stable. This result differs drastically from the results of our robotic simulations in which this transition never fully occurred (Mann-Whitney U test on the proportion of stags hunted successfully during the last generation, *p*-value <0.001).

### Starting with a random initial population

**Fig 5:**
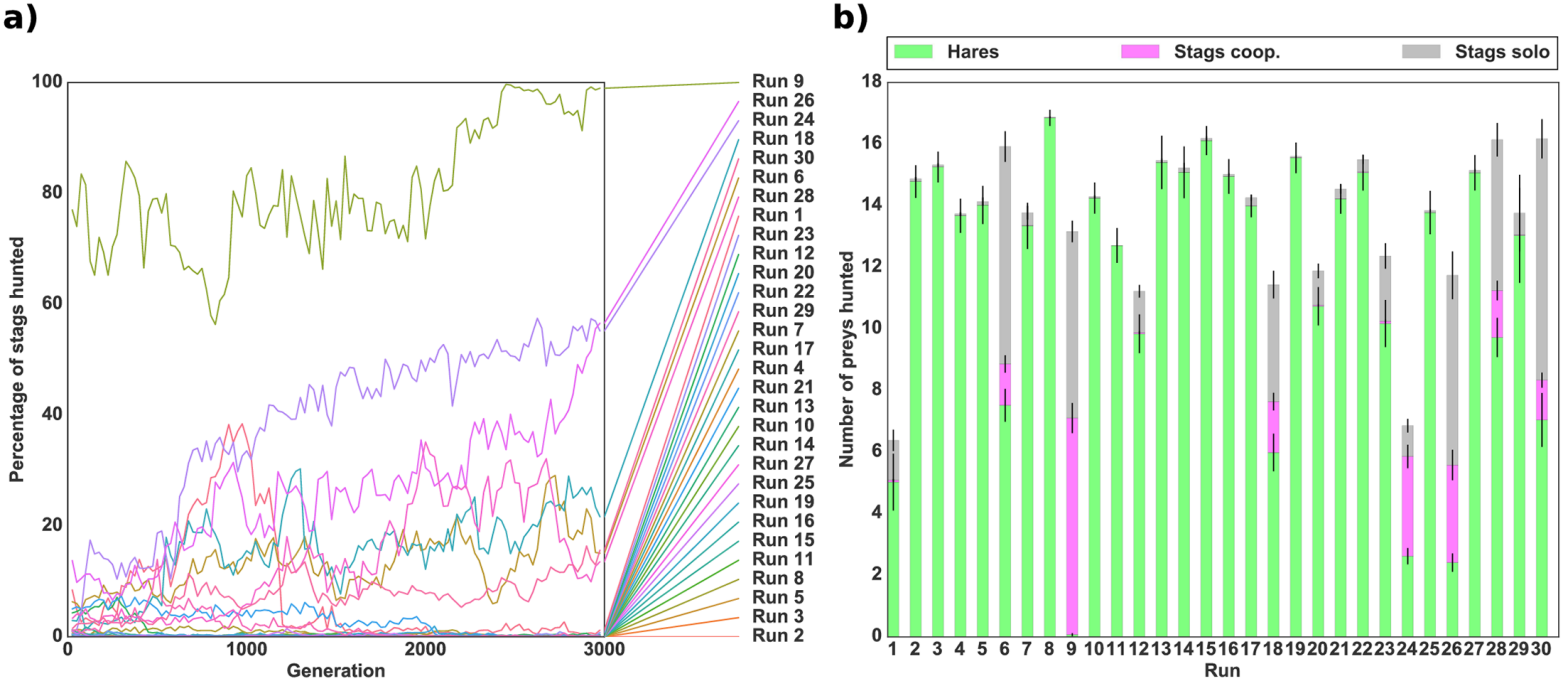
Evolution of cooperation with no initial hunting strategy. *(a)* Evolution of the mean percentage of stags hunted successfully (i.e. cooperatively) with respect to the total number of prey hunted in a robotic simulation. *(b)* Mean number of prey hunted during the last generation of evolution for each independent run. The bottom green bar represents the number of hares hunted, the middle pink bar the number of stags hunted successfully (cooperatively) and the top grey bar the number of failed hunts (stags hunted alone). The standard deviation for each quantity is shown by black lines. Rewards were 50 for a hare, 0 for a stag hunted alone, and 500 for a stag hunted cooperatively, as presented in Table 1. The number of prey (18) was kept constant throughout the simulation by replacing killed prey by a prey of the same type.

In a second experiment, we wanted to investigate the evolution of hunting strategies “from scratch”, with the individuals’ genotypes initialized with random values, rather than evolved with a specific hunting strategy. Fig 5 shows the mean percentage of stags hunted over time and the mean number of prey hunted during the last generation. We observed the transition to a clearly cooperative strategy in a single run, while in two other runs, 50% of prey hunted were stags. In the 27 remaining runs the proportion of stags hunted was less than 25%. In comparison, in simulations using the standard game-theoretic version of the stag hunt where individuals are initially unable to hunt, stag hunting evolved and remained stable in every run (see supporting information S1 Fig).

The above experiments show that mechanistic constraints have a critical effect on the evolution of coordinated collective actions. In a simple game-theoretic analysis in which the hunting strategy is encoded by a single binary gene, collective behaviour systematically evolved. However, in a setting where the hunting strategy was determined by a more complex artificial neural network, cooperative behaviour evolved in fewer than 10% of cases. These results encourage further exploration into the evolutionary origin of coordinated collective actions and the mechanisms which may facilitate their evolution. In the following section, we explore two such mechanisms.

### When stags can be hunted alone

In the next experiment, food was also rewarded for hunting a stag in a solitary fashion so that cooperative behaviour did not entail a risk. We wanted to study whether hunting a stag alone could act as a transition towards the evolution of the collective strategy. Hunting a stag alone was given the same reward as hunting a hare (Table 3), differing from classical models of the stag hunt.

**Table 3:** Food rewards for hunting different prey.

Prey		Food Reward
Hare	*alone*	50
	*coop.*	50
Stag	*alone*	50
	*coop.*	500

The reward depends on whether these prey were hunted alone or cooperatively. There is a reward for stags hunted alone in this case.

**Fig 6:**
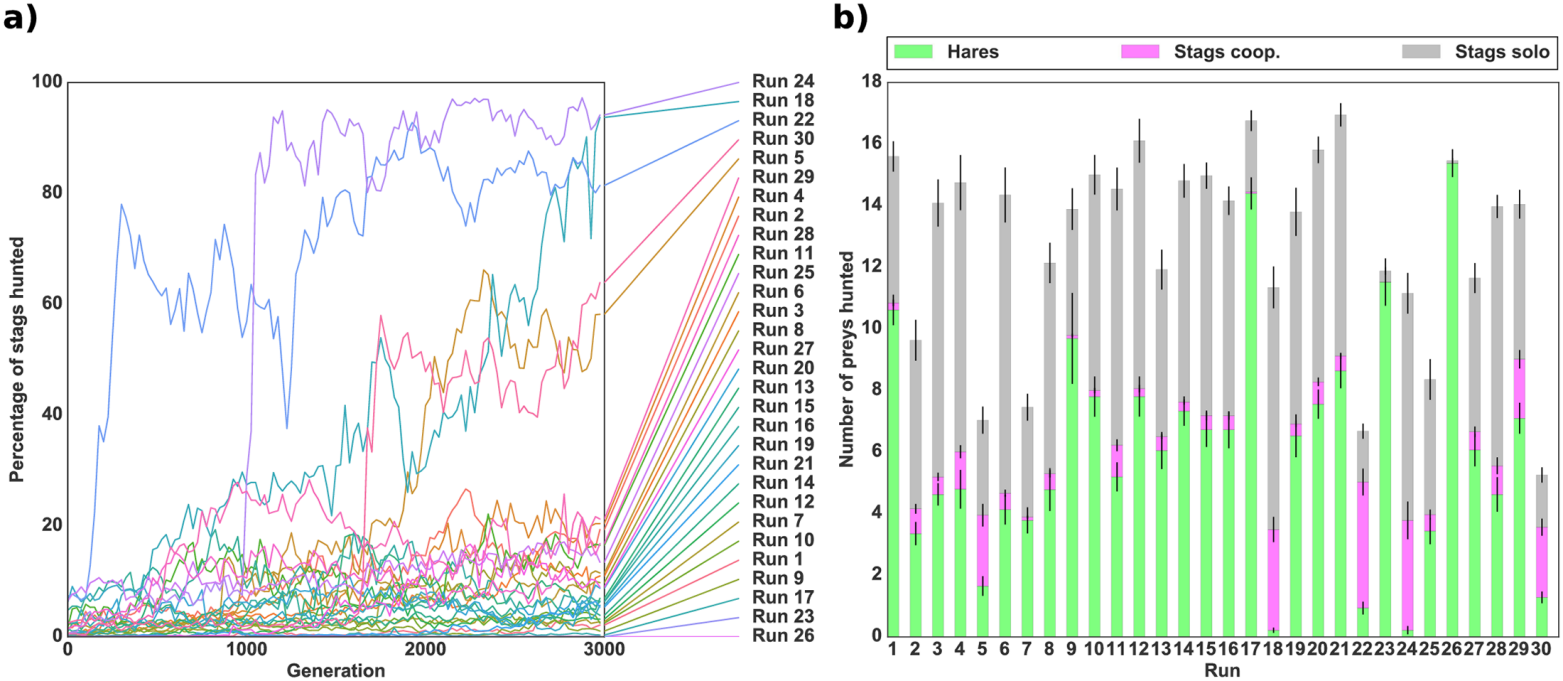
Evolution of cooperation with an initial hare-hunting strategy and a reward for solitary stag hunting. *(a)* Evolution of the mean percentage of stags hunted successfully (i.e. cooperatively) with respect to the total number of prey hunted in a robotic simulation. *(b)* Mean number of prey hunted during the last generation of evolution for each independent run. The bottom green bar represents the number of hares hunted, the middle pink bar the number of stags hunted cooperatively and the top grey bar the number of stags hunted alone. The standard deviation for each quantity is shown by black lines. The population for each of the 30 independent runs was previously evolved in an environment with only hares. Rewards were 50 for a hare, 50 for a stag hunted alone, and 500 for a stag hunted cooperatively as presented in Table 3. The number of prey (18) was kept constant throughout the simulation by replacing killed prey by a prey of the same type.

Fig 6 shows the results of robotic simulations where individuals initially evolved to hunt hares (as in Fig 3). As expected, the evolution of collective hunting was significantly facilitated when the risk of hunting stags alone was removed (Mann-Whitney, *p*-value <0.001). The populations completely switched to hunting stags in two runs out of 30, and in three other runs, more than 50% of the prey hunted were stags, with a large part of the prey hunted cooperatively in each of these runs. However, in most of the runs (25 out of 30), the evolved strategy was to hunt both types of prey in a solitary fashion. From these results, it entails that the individuals are still hindered by the evolution of a successful coordination strategy.

### The role of genetic relatedness

Genetic relatedness among social partners is known to influence the evolution of many types of social traits [1]. In particular, [6] showed how it can facilitate the evolution of cooperation in a stag hunt game [6, chapetr 3]. It can yield more frequent interaction between cooperators, which in turn increases their probability of benefiting from cooperative behaviour. In order to include this mechanism, we considered an extreme situation in which each individual is always paired with a clone of itself, known as “clonal selection” in robotics, ensuring a maximal genetic relatedness of 1.

**Fig 7:**
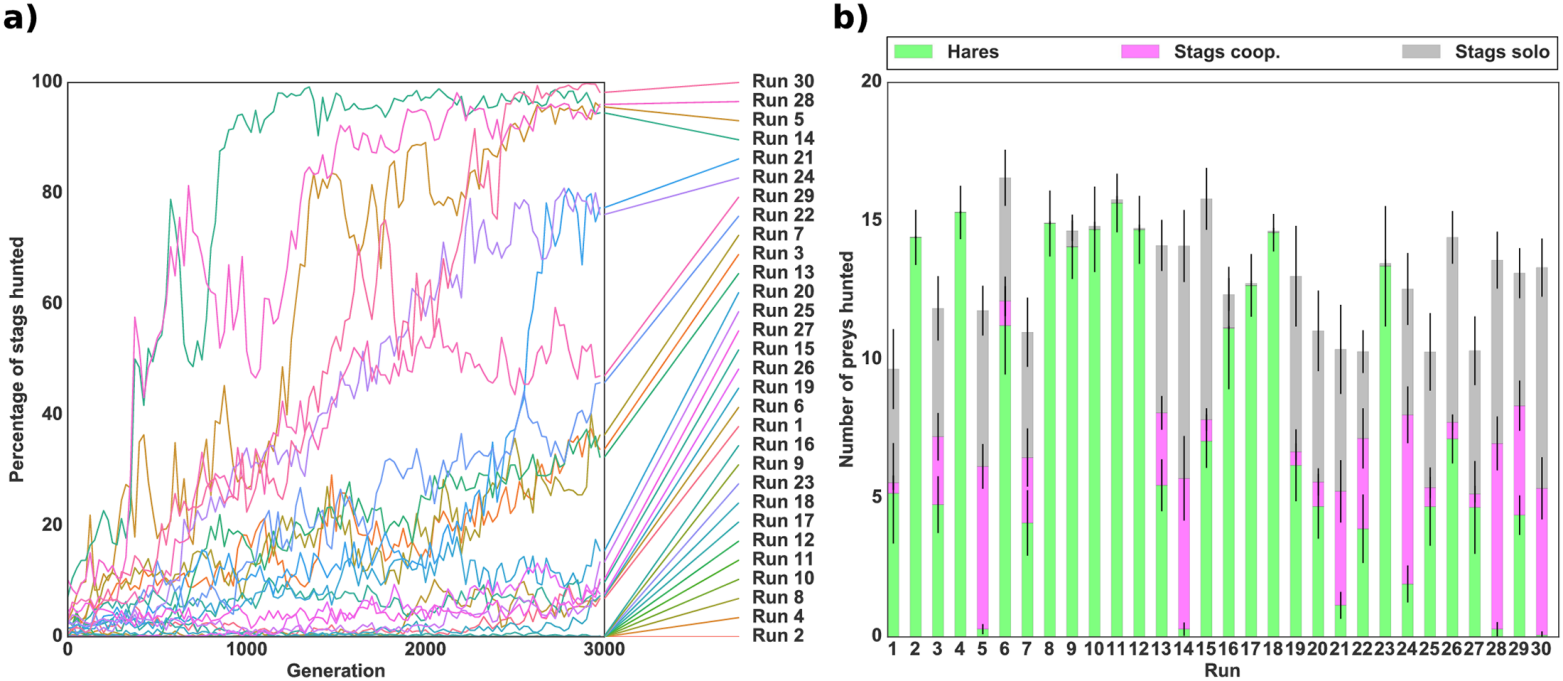
Evolution of cooperation under maximal genetic relatedness with an initial hare-hunting strategy. *(a)* Evolution of the mean percentage of stags hunted successfully (i.e. cooperatively) with respect to the total number of prey hunted in a robotic simulation. *(b)* Mean number of prey hunted during the last generation of evolution for each independent run. The bottom green bar represents the number of hares hunted, the middle pink bar the number of stags hunted successfully (cooperatively) and the top grey bar the number of failed hunts (stags hunted alone). The standard deviation for each quantity is shown by black lines. The population for each of the 30 independent runs was previously evolved in an environment with only hares. The genetic relatedness between paired individuals was 1. Rewards were 50 for a hare, 0 for a stag hunted alone, and 500 for a stag hunted cooperatively as presented in Table 1. The number of prey (18) was kept constant throughout the simulation by replacing killed prey by a prey of the same type.

These results show that genetic relatedness has a positive effect on the evolution of cooperation (Fig 7). In four out of 30 runs the population evolved the cooperative strategy. Moreover, in two other runs, stags accounted for more than 75% of prey hunted, as compared to less than 25% without relatedness (Mann-Whitney, *p*-value <0.005). When the initial population was random, rather than only hare hunters (see supporting information S2 Fig), the positive effect of genetic relatedness was also observed in 12 out of 30 runs, where more than 50% of prey hunted were stags.

## Discussion

There is a profound difference between evolutionary game-theoretic and robotic simulations of the stag hunt. Using identical model parameters, the transition from the solitary equilibrium to the social equilibrium always occurred in game-theoretic simulations, but was extremely unlikely in robotic simulations, occurring in 1 run out of 30. The complexity of the mapping between genotype and phenotype is responsible for much of this contrast. Individuals involved in a coordination game such as the stag hunt face a chicken & egg problem: the cooperative behaviour must be beneficial in order to evolve, but no individual can benefit from this behaviour unless the behaviour is already expressed by other individuals. When binary variation at a single genetic locus encodes the expression of the solitary or cooperative strategy, a single mutation is sufficient for a cooperative mutant to appear in a resident population of solitary individuals. In a finite population, demographic stochasticity can then lead to the rise of cooperators above the invasion threshold, at which point natural selection leads to their fixation, switching from a solitary equilibrium to a social one. In contrast, in our robotic simulations, the mapping between genotype and phenotype is more complex. Adopting the social strategy entails both a modification of the preferred hunting target and the ability to coordinate with others. Thus, several mutations are necessary for the appearance of full-fledged cooperative behaviour. As several individuals must carry these multiple mutations for the behaviour to become beneficial, the transition to the cooperative equilibrium is nearly impossible.

**Fig 8:**
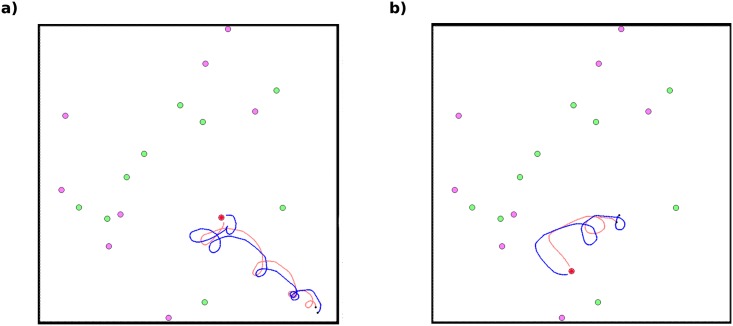
Snapshots of a simulation after two hunts. In each of these snapshots, we show the path travelled by each hunter (in different colours) since their last prey was hunted. The black dots represent the positions of the hunters at their last kill.

In particular, in our robotic simulations we were able to observe that coordination entails a specific and rather complex behaviour. Fig 8 (see also supporting information S1 Movie) shows the behaviours evolved by the best individuals in the cooperative run shown in Fig 5 (Run 9). The solution they evolved for coordination was to circle around one another, allowing each of them to constantly see their partner while both moving closer to a stag. This behaviour was replicated in every cooperative run. We thus observed the evolution of an ingenious (given the agents’ limited capabilities) and complex hunting strategy. These findings demonstrate that the practical mechanics of behaviour can have important evolutionary consequences, and that models which ignore these properties may lead to misleading predictions.

Moreover, the evolution of cooperation is also strongly impacted by ecological features. Social hunting poses a bootstrapping problem because it entails both a modification of the preferred hunting target and an ability to coordinate with others. Its evolution can be facilitated, therefore, if hunters have a reasonable probability of hunting the same prey as their partner, just by chance, with no need of active coordination. Biologically, this could occur if hunters live in a dense social environment (with many other hunters in the vicinity), and/or if the density of prey is low, such that the likelihood of ending up on the same prey is large. To test this possibility, we conducted additional experiments where the density of prey was varied. The number of prey was whether (1) decreased from 18 to 6 or (2) increased from 18 to 30. The population was initially constituted of hare hunters and we kept the same ratio of prey as in previous experiments (i.e. 50% of hares and 50% of stags). We show (see supporting information S3 Fig(a)) that when the number of prey is decreased (6) the transition to a cooperative strategy is facilitated (Mann-Whitney, *p*-value <0.05) as in 9 runs out of 30, more than 30% of the prey hunted are stags. In comparison, a higher density of prey (30) entails that it is impossible to evolve cooperation (see supporting information S3 Fig(b)). These results reinforce our claim that the practical mechanics of coordination are crucial in understanding the evolution of cooperation. In particular, here, the precise ecological situation faced by individuals plays a key role in the transition to the collective equilibrium.

Finally, the complexity of coordination suggests that the recycling of a previously evolved trait could be necessary for the transition to cooperation, i.e. individuals could coordinate thanks to behavioural features that may not have been selected for cooperation at first. Such features could include the evolution of communication, or a leader-follower strategy. The role of both of these behaviours has already been studied in real-life stag hunt type interactions in chimpanzees and human children [25, 26], and there is an already extensive literature in evolutionary robotics on their role in the evolution of collective actions [16, 19, 22, 27]. This offers some directions for future works on this problem.

## Supporting Information

S1 Fig Evolution of cooperation in a game-theoretic simulation with an initial hare-hunting strategy. Evolution of the mean percentage of stags hunted with respect to the total number of prey hunted where individuals are initially unable to hunt for 30 independent runs. Rewards were 50 for a hare, 0 for a stag hunted alone, and 500 for a stag hunted cooperatively.
(EPS)

S2 Fig Evolution of cooperation under maximal genetic relatedness with no initial hunting strategy. Evolution of the mean percentage of stags hunted with respect to the total number of prey hunted in a robotic simulation. The genetic relatedness between paired individuals was 1. Rewards were 50 for a hare, 0 for a stag hunted alone, and 500 for a stag hunted cooperatively as presented.
(EPS)

S3 Fig Evolution of cooperation with an initial hare-hunting strategy and a varied density of prey. Evolution of the mean percentage of stags hunted successfully (i.e. cooperatively) with respect to the total number of prey hunted in a robotic simulation when the number of prey was *(a)* 6 and *(b)* 30. The population for each of the 30 independent runs was previously evolved in an environment with only hares. Rewards were 50 for a hare, 0 for a stag hunted alone, and 500 for a stag hunted cooperatively as presented in Table 1. The number of prey was kept constant throughout the simulation by replacing killed prey by a prey of the same type.
(EPS)

S1 Movie Movie simulation of the best individual in a cooperative run. Complete trial of the best individual in a run where a cooperative behaviour evolved. Rewards were 50 for a hare, 50 for a stag hunted alone, and 500 for a stag hunted cooperatively. The left section of the movie shows the behaviours of the simulated robots during the trial while the right section is a representation of the sensory information of each hunter. The sensory information of the agent circled in black is shown on the top part of this latter section and the sensors of the other agent on the bottom. For each agent, the top twelve rectangles represent the type of agent recognized by each ray of the camera. The activation of each proximity sensors is shown below, with the length of each ray representing the strength of activation (i.e. the proximity to an obstacle) and the blue circle representing the radius of maximum activation.
(MP4)

S1 Code Source code. (GZ)
