# Supplementary material for: To Cooperate or Not to Cooperate: Why Behavioural Mechanisms Matter
Source: PLoS Comput Biol. 2016 May 5;12(5):e1004886. doi: 10.1371/journal.pcbi.1004886 (PMC4858277; doi:10.1371/journal.pcbi.1004886)
Supplement: S1 Code — (GZ) [file pcbi.1004886.s005.gz › StagHuntExperiments/RoboticExperiments/doc/old/html/ref.html]

xml version="1.0" encoding="utf-8"?


Sferes2 – reference


# Sferes2 – reference

{back to main page}

## Table of Contents

- 1 The Sferes2 framework 
  - 1.1 Main concepts 
    - 1.1.1 Static object-oriented paradigm
    - 1.1.2 Static parameters
    - 1.1.3 Macros and special functions 
      - 1.1.3.1 Classes
      - 1.1.3.2 Parameter handling
  - 1.2 Mastering boost
  - 1.3 UML Diagram
  - 1.4 Overview of the call graph
- 2 Main classes and files 
  - 2.1 Naming conventions
  - 2.2 Fitness 
    - 2.2.1 Fitness
    - 2.2.2 Defining your own fitness
    - 2.2.3 View mode
  - 2.3 Evolutionary algorithms 
    - 2.3.1 Shared parameters
    - 2.3.2 RankSimple
    - 2.3.3 Nsga2
    - 2.3.4 EpsMOEA
    - 2.3.5 Defining your own EA
  - 2.4 Statistics 
    - 2.4.1 BestFit
    - 2.4.2 MeanFit
    - 2.4.3 ParetoFront
    - 2.4.4 Defining your own statistics
  - 2.5 Genotypes 
    - 2.5.1 BitString
    - 2.5.2 EvoFloat
    - 2.5.3 Defining your own genotype
  - 2.6 Phenotypes (Individuals) 
    - 2.6.1 Indiv
    - 2.6.2 Parameters
    - 2.6.3 Defining your own phenotype
  - 2.7 Evaluators 
    - 2.7.1 Eval
    - 2.7.2 Parallel
    - 2.7.3 Mpi
    - 2.7.4 Defining your own evaluator
  - 2.8 Modifiers 
    - 2.8.1 Dummy
  - 2.9 Misc
- 3 Modules 
  - 3.1 NN
  - 3.2 Fastsim
  - 3.3 Python
- 4 Writing modules

## 1 The Sferes2 framework

### 1.1 Main concepts

#### 1.1.1 Static object-oriented paradigm

Object-oriented programming (OOP) has many advantages which are
desirable for complex high-level code. However, classic OOP (e.g. in
C++) heavily relies on virtual methods to implement abstraction and
polymorphism. Such an approach has a significant run-time overhead:

- selecting the good method to call adds an indirection (the software
  have to check the virtual table)
- abstract/virtual methods cannot be inlined, resulting in a overhead
  for very simple methods (e.g. setters/getters).

In a few word, we want the main benefits of OOP (abstraction,
re-usability) and the efficiency of low-level languages.

One solution in C++ is to use the "curiously recurring template
pattern"
(http://en.wikipedia.org/wiki/Curiously\_Recurring\_Template\_Pattern). Here
is the idea:

```
template <class Derived> struct Base
{
    void interface()
    {
        static_cast<Derived*>(this)->implementation();
    }
    void f()
    {
       for(int i = 0; i < 3; ++i) interface();
    }
};
 
struct Derived : Base<Derived>
{
    void implementation() { std::cout<<"impl"<<std::endl; }
};
```

This code mimics a virtual call of an abstract method: in the abstract
class (Base), a generic algorithm can use methods which are only
defined in the derived classes. Extending this pattern to draw a full
hierarchy of classes requires more work; sferes2 relies on the method
described in the following paper:

Nicolas Burrus, Alexandre Duret-Lutz, Thierry Géraud, David Lesage
and Raphaël Poss. A Static C++ Object-Oriented Programming (SCOOP)
Paradigm Mixing Benefits of Traditional OOP and Generic
Programming. In *Proceedings of the Workshop on Multiple Paradigm with OO Languages (MPOOL'03)*, 2003.
http://www.lrde.epita.fr/dload/papers/mpool03.pdf

This mechanics is hidden in sferes2 behind some macros (see section macros)

#### 1.1.2 Static parameters

Evolutionary algorithms have a lot of parameters (e.g. population
size, mutation rate, etc.) and we need a convenient way to set them. A
configuration file (e.g. an XML file) is often used; however, such a
method has two drawbacks:

- some code to read the files has to be written and kept synchronized
  with the objects;
- parameters are unknown at compile time so some checks (e.g. if
  (mutation\_type = x) {} else { } …) have to be done many times
  whereas they are useless.

In sferes2, parameters are defined at compile time using a structure
which contains only constants. This structure is passed to all sferes2
classes so they can access to the parameters. This method allows to
avoid to write read/write code for parameters. It also allows the
compiler to propagate constants and settings in the whole source code,
resulting in an executable optimized for the specific parameters.

#### 1.1.3 Macros and special functions

To simplify the writing of "static virtual functions" and the
definition of static parameters, sferes2 provides several macros which
are defined in stc.hpp.

##### 1.1.3.1 Classes

- SFERES\_CLASS(Name): defines the base class Name ; this class uses
  the "static" inheritance scheme and takes a Param template parameter.
- SFERES\_CLASS\_D(Name, Parent): derives the class Name from Parent
- SFERES\_PARENT(Name, Parent)::method(): calls method() in the parent class
- stc::exact(this)->method(): calls an abstract method() which is
  defined in the derived classes

Moreover, sferes2 provides special macros to define specific classes
(SFERES\_FITNESS, SFERES\_INDIV, SFERES\_EA, etc.). See ref.

Example:

```
SFERES_CLASS(X)
{
  X() { std::cout<<"X"<<std::endl; }
  void test() { stc::exact(this)->blah(); }
  void test2() { std::cout<<"test2"<<std::endl; }
};
// Y derives from X
SFERES_CLASS_D(Y, X)
{
  Y() { std::cout<<"Y"<<std::endl; }
  void blah() 
  { 
    std::cout<<"param::test::x"<<Param::test::x<<std::endl;
    SFERES_PARENT(Y, X)::test2();
  }
};
// Z derives from Y
SFERES_CLASS_D(Z, Y)
{
  Z() { std::cout<<"Z"<<std::endl; }
  void blah() 
  { 
    std::cout<<"Z"<<std::endl;
  }
};
// sample parameter (see next subsection)
struct Params_test
{
  struct test
  {
    static const float x = 0.1f;
  };
};
// to instantiate Y and Z (X is abstract):
int main()
{
  // Y uses a parameter
  Y<Param_test> y;
  // Z doesn't use any parameter, these two forms are possible:
  Z<> z1;
  Z<Param_test> z2;
  return 0;
}
```

##### 1.1.3.2 Parameter handling

The basic parameter format employs static const members in nested
structures. For instance:

```
struct Param
{
  struct pop
  {
    static const size_t size = 100;
    static const float x = 0.2f;
  };
};
```

However, c++ does not allow to define static const string or arrays in
these structures. To solve this problem, sferes2 provides two macros:

- SFERES\_ARRAY(type, name, val1, val2, …)
- SFERES\_STRING(name, "string")

```
struct Param
{
  struct test
  {
    SFERES_ARRAY(float, my_array, 1.0f, 2.0f, 3.0f);
    SFERES_STRING(my_string, "hello world");
  };
};
```

These parameters can then be retrieved in the source code in the
following way:

```
...
// size
size_t s = Params::test::my_array_size();
// 2nd element
float v = Param::test::my_array(2);
// string
const char* c = Params::test::my_string();
```

### 1.2 Mastering boost

Sferes2 heavily relies on boost libraries (see
http://www.boost.org). To implement your own
algorithms in the sferes2 framework, you should be familiar enough
with the following libraries:

- boost::shared\_ptr:
  http://www.boost.org/doc/libs/1400/libs/smartptr/sharedptr.htm
- boost::serialization (useful to define your own genotypes and
  statistics):
  http://www.boost.org/doc/libs/1400/libs/serialization/doc/index.html

The following libraries are used in sferes2 but you might never notice
them:

- BOOST\_FOREACH (to simplify loops): http://www.boost.org/doc/libs/1400/doc/html/foreach.html
- boost::filesystem (to create directories, etc.): http://www.boost.org/doc/libs/1400/libs/filesystem/doc/index.htm
- boost::fusion (list of statistics):
  http://www.boost.org/doc/libs/1400/libs/fusion/doc/html/index.html
- boost::mpi (MPI evaluator): http://www.boost.org/doc/libs/1400/doc/html/mpi.html

### 1.3 UML Diagram

Here is a "conceptual" UML diagram of the sferes2 framework (modules
are not represented). It's "conceptual" because some abstract classes
are not implemented (because they are useless from an implementation
point of view) but should exist in the mind of the user. Moreover,
each class is parametrized by the Param class (see previous section),
which is not represented on the diagram.

### 1.4 Overview of the call graph

On this simplified call graph:

- ea\_t : type of your EA (e.g. ea::Nsga2)
- eval\_t : type of your evaluator (e.g. eval::Parallel)
- gen\_t : type of your phenotype (e.g gen::EvoFloat)
- modifier\_t : type of your modifier (e.g. modif::Dummy)
- phen\_t : type of your phenotype (e.g. phen::Parameters)
- stat\_t : type of your statistics vector
  (e.g. boost::fusion::vector<stat::BestFit<phen\_t> > )

Time flows from left to right (i.e. random\_pop() is called before
epoch() and so on). Methods with a double bar are called for each individual or
each new individual.

## 2 Main classes and files

### 2.1 Naming conventions

- Names representing **classes** must be in mixed case starting with upper
  case: MyClass
- **Variable and method names** must be in lower case, using underscores to
  separate words: my\_variable, my\_method()
- Names of **protected and private members** must start with an underscore:
  \_my\_private\_member, \_my\_private\_method()
- **File names** must be in lower case, using underscores to separate
  words. A file which contains a class MyClass should be put in a file
  my\_class.hpp
- **File structure** mirrors namespace structure. For instance
  gen::MyClass is in the file gen/my\_class.hpp
- **Named constants** (including enumeration values) must be all uppercase
  using underscores to separate words
- **Getters** should have the name of the attribute. For instance,
  this->\_objs should be accessed using this->objs()
- **Setters** should start with "set\_" followed by the name of
  the attribute. For instance: set\_objs(const std::vector<float>& ov)
- The **public section** should be the first section of a class
- Type names defined using **typedefs** should end with \_t (e.g. iterator\_t)

Full example:

```
// filename: sferes/fit/my_test.hpp
namespace sferes
{
  namespace fit
  {
    class SFERES_CLASS(MyTest)
    {
    public:
      typedef float f_t;
      MyTest() {}
      void my_method() { _my_impl(); }
      float my_attr() const { return _my_attr;}
      float set_my_attr(float a) { _my_attr = v; }
    protected:
      float _my_attr;
      void _my_impl() {}
    };
  }
}
```

### 2.2 Fitness

#### 2.2.1 Fitness

- **File:** sferes/fit/fitness.hpp
- **Description:** Fitness is the base class of fitness functions in
  sferes2. It has two main attributes:
  - float \_value (setter/getter Fitness::value()) -> the value of the fitness for
    single-objective optimization
  - std::vector<float> \_objs (setter/getter Fitness::objs()) -> the value of
    each objective, for multiobjective optimization
- This class is abstract

#### 2.2.2 Defining your own fitness

In most sferes2 experiments, you will have to design your own fitness. A
fitness should:

- be defined using SFERES\_FITNESS(MyFitness, sferes::fit::Fitness)
- define the eval() method, which takes an individual
- attribute a fitness value in this->\_value (single objective
  optimization) or this->\_objs (multiobjective optimization)

  A fitness is a "normal" class and consequently you can
  add other methods or attributes to suit your needs.

Example:

```
// single objective fitess
SFERES_FITNESS(FitnessSingle, sferes::fit::fitness)
{
  // (optional) constructor
  FitnessSingle()
    {
      this->_value = -1;
    }
  // evaluation
  template<typename Indiv>
    void eval(Indiv& indiv)
  {
    this->_value = -42;
  }
};

// two-objective fitness
SFERES_FITNESS(FitnessMulti, sferes::fit::fitness)
{
  // constructor
  FitnessMulti()
    {
      this->_objs.resize(2);   
    }
  // evaluation
  template<typename Indiv>
    void eval(Indiv& indiv)
  {
    this->_objs[0] = -42;
    this->_objs[1] = 42;
  }
};
```

#### 2.2.3 View mode

When loading a result file, it is often useful to slightly change the
fitness function, for instance to display/log data about the behavior
of the individual. This can be easily done in sferes2 using the
boolean Fitness::mode() than can takes two values:

- fit::mode::view when you are viewing an individual via –load
- fit::mode::eval when you are evaluting individuals (during the
  evolutionary process)

Example:

```
// single objective fitess
SFERES_FITNESS(FitnessSingle, sferes::fit::fitness)
{
  // evaluation
  template<typename Indiv>
    void eval(Indiv& indiv)
  {
    if (this->mode() == sferes::fit::mode::view)
      {
      std::ofstream ofs("fit.dat");
      ofs<<"this is a log file !"<<std::endl;
      }
    this->_value = -42;
  }
};
```

### 2.3 Evolutionary algorithms

**Warning** Evolutionary algorithms *maximize* the fitness (whereas most
optimization algorithms minimize the cost function).

#### 2.3.1 Shared parameters

All evolutionary algorithms (EA) use the following parameters:

- static const size\_t Params::pop::size = population size
- static const float Params::pop::initial\_aleat = during the random generation,
  Params::pop::initial\_aleat × Params::pop::size individuals are
  generated and only the Params::pop::size best ones are kept
- static const int Params::pop::dump\_period: the period between each write of results, examples:
  - 1: statistics are written at each generation (gen\_0, gen\_1, gen\_2, …)
  - -1: statistics are never written (useful for unit tests)
  - 10: statistics are written each 10 generations (gen\_0, gen\_10, …)

#### 2.3.2 RankSimple

- **File:** sferes/ea/rank\_simple.hpp
- **Unit test:** tests/ea/rank\_simple.cpp
- **Typical typename:**:

```
typedef sferes::ea::RankSimple<phen_t, eval_t, stat_t, modifier_t, Params> ea_t;
```

- **Description:** A rank-based single-objective evolutionary
  algorithm. Individuals are selected using the following formula:
  - n = kr × p
  - κ = cn + 1 - 1
  - f = n / (κ + 1)
  - i = p - f × log(r × κ + 1)
  - where
    - c = Params::pop::coeff
    - kr = Params::pop::keep\_rate
    - p = Params::pop::size
    - r = a random number in [0,1[
    - i = index of the selected individual
- parameters:
  - static const float Params::pop::coeff = a internal parameter (see the previous
    formula) ; typical value : 1.1
  - static const float Params::pop::keep\_rate = proportion of individuals kept from a
    generation to the next one ; typical value : 0.2 to 0.6

#### 2.3.3 Nsga2

- **File:** sferes/ea/nsga2.hpp
- **Unit test:** tests/ea/nsga2.cpp
- Example: examples/ex\_nsga2.cpp
- **Typical typename:**:

```
typedef sferes::ea::Nsga2<phen_t, eval_t, stat_t, modifier_t, Params> ea_t;
```

- **Description:** Implementation of the NSGA-II multiobjective
  evolutionary algorithm. See:
  - Deb, K. (2001). Multi-Objective Optimization Using Evolutionary
    Algorithms. *Wiley*.
  - Deb, K., Pratap, A., Agarwal, S. and Meyarivan, T. (2002). A fast
    and elitist multiobjective genetic algorithm: NSGA-II. In *IEEE transactions on evolutionary computation*, 6:2:182-197
- **Parameters:** pop\_size must be divisible by 4
- **Note:** NSGA-II can be efficient for single-objective optimization as
  it corresponds to a classic tournament-based evolutionary algorithm. In
  sferes2, a 1-sized \_objs vector in the fitness can be employed (don't
  use value).

#### 2.3.4 EpsMOEA

- **File:** sferes/ea/eps\_moea.hpp
- **Unit test:** tests/ea/eps\_moea.cpp
- **Typical typename:**:

```
typedef sferes::ea::EpsMOEA<phen_t, eval_t, stat_t, modifier_t, Params> ea_t
```

- **Description:** Implementation of the ε-MOEA multiobjective
  evolutionary algorithm. See:
  - Deb, K., Mohan, M. and Mishra, S. (2005). Evaluating the
    ε-domination based multi-objective evolutionary
    algorithm for a quick computation of pareto-optimal solutions. In
    *Evolutionary Computation*, 13:4:501-525.
- Parameters
  - SFERES\_ARRAY(float, eps, 0.0075f, 0.0075f) = values of ε
    for each objective. There should be as many values as objectives.
  - SFERES\_ARRAY(float, min\_fit, 0.0f, 0.0f) = minimum value of
    fitness for each objective.There should as many values as objectives
  - static const size\_t grain = ε-MOEA is a
    steady-state algorithm which adds individuals one by one. A basic
    implementation would prevent the parallelization of individuals'
    evaluation. The parameter grain allows to generate and evaluate
    *grain* indidividuals in paralell at each generation. Typical value
    is size / 4.
- **Notes:**
  - ε-MOEA employs ε-dominance to sort
    individuals. This allows users to tune the precision of the pareto
    front for each objective and often results in better performance
    than NSGA-II.
  - EpsMOEA should not be used with a modifier (because it uses an
    archive). You should always use fit::ModifierDummy

#### 2.3.5 Defining your own EA

To define your own EA, you should use the macro SFERES\_EA(Class,
Parent). Mandatory methods:

- random\_pop(): fill this->\_pop with random individuals
- epoch(): main loop of the algorithm

Defined types (these types will be available in your class):

- Phen: phenotype
- Eval: evaluator
- Stat: statistics vector
- FitModifier: modifier
- Params: parameters

```
SFERES_EA(Test, Ea)
{
 public:
 Test() {}
 void random_pop()
 { /* your code */ }
 void epoch()
 { /* your code */ }
};
```

Multiobjective EA must also define a pareto\_front() method, see ParetoFront.

### 2.4 Statistics

Statistics should be combined in a boost::fusion::vector (see:
http://www.boost.org/doc/libs/1400/libs/fusion/doc/html/fusion/container/vector.html)
before being passed to a EA. For instance:

```
typedef boost::fusion::vector<stat::BestFit<phen_t, Params>, stat::MeanFit<Params> >  stat_t;
```

(yes, it is a vector of *typenames*).
You can put in the vector as many statistics types as you
desire.

#### 2.4.1 BestFit

- **File:** sferes/stat/best\_fit.hpp
- **Typical typename:**

```
typedef boost::fusion::vector<sferes::ea::BestFit<phen_t, Params> > stat_t;
```

- **Description:**
  - stores the individual with the highest fitness in \_best (getter:
    BestFit::best() ). *This assumes that the population is sorted* and
    consequently this statistics stores the first individual of the
    population.
  - This class is designed for single objective optimization and
    consequently works using Fitness::\_value (and not Fitness::\_objs)
  - writes the value of the best fitness in the file
    <result\_directory>/bestfit.dat ; this file should be easily plotted
    with gnuplot or R.

#### 2.4.2 MeanFit

- **File:** sferes/stat/mean\_fit.hpp
- **Typical typename:**

```
typedef boost::fusion::vector<sferes::ea::MeanFit<phen_t, Params> > stat_t;
```

- **Description:** computes the mean fitness (single objective, based on
  Fitness::\_value). This class is mainly aimed at providing a simple
  example of statistics.

#### 2.4.3 ParetoFront

- **File:** sferes/stat/pareto\_front.hpp
- **Typical typename:**

```
typedef boost::fusion::vector<sferes::ea::ParetoFront<phen_t, Params> > stat_t;
```

- **Description:** stores the set of non-dominated individuals by copying
  the list returned by ea\_t::pareto\_front(). ParetoFront does not
  compute the pareto front; this class assumes that the EA computed
  it. This implies that multiobjective EA classes should define the following method:

```
SFERES_EA(MyEA, Ea)
{
 public:
  std:vector<Indiv> &pareto_front() const { /* your code */ }
};
```

- **Note:** since this statistic can store many individuals, to load a
  result file you must give the identifier (the rank in the Pareto
  front) to your executable. For instance, assuming that ParetoFront
  in the first (0th) statistics in the stat vector, loading the 10th
  individual should look like:

```
./my_exp --load gen_100 -s 0 -n 10 -o output
```

#### 2.4.4 Defining your own statistics

```
SFERES_STAT(MyStat, Stat)
{
  // examines the population (via the EA) to update the statistics
  template<typename E> void refresh(const E& ea) { /* your code */ }
  // writes the content of the statistics in the stream; k is the number
  // of the individual in the statistics (e.g. in a Pareto front, the
  // statistics contains many individuals)
  void show(std::ostream& os, size_t k) const { /* your code */ }
  // serializes the statistics using boost::serialization
  template<class Archive> void serialize(Archive & ar, const unsigned int version)
  { /* your code */ }
}
```

### 2.5 Genotypes

#### 2.5.1 BitString

- **File:** sferes/gen/bit\_string.hpp
- **Unit test:** sferes/gen/bit\_string.hpp
- **Description:** vector of bitstrings which can be used to represent a
  vector of integer parameters or a vector of discrete
  parameters. Cross-over is a simple one point cross-over (it always
  happens). The data can be easily converted to int (in the range
  0..2b, where b is the number of bits for each bitstring) via
  BitString<>::int\_data(i) or to float (in the range 0..1) via
  BitString<>::data(i)
- **Template parameter:** the number of bitstrings
- **Parameters (struct bit\_string):**
  - static const size\_t nb\_bits -> number of bits for each bitstring
  - static const float mutation\_rate -> the mutation rate for a
    bitstring (the bitstring i is mutated if a random real is belows mutationrate)
  - static const float mutation\_rate\_bit -> the mutation rate *for each bit*. In pseudo-code, here is the mutation procedure:

```
foreach(bitstring b in genotype)
  if (rand(0, 1) < Params::bit_string::mutation_rate)
    foreach(bit i in b)
      if (rand(0, 1) < Params::bit_string::mutation_bit)
        flip(i)
```

- **Typical parameters:**

```
struct Params
{
  struct bit_string
  {
    static const size_t nb_bits = 8;
    static const float mutate_rate = 0.1f;
    static const float mutate_rate_bit = 0.1f;    
  };
};
```

- **Typical typename:**

```
typedef BitString<10, Params> gen_t;
```

#### 2.5.2 EvoFloat

- **File:** sferes/gen/evofloat.hpp
- **Unit test:** sferes/tests/gen/evo\_float.cpp
- **Description:** This genotype is a n-sized vector of real (float)
  numbers. It is designed for real optimization.
- Three mutation types are available:
  - polynomial: polynomial mutation, as defined in Deb(200)1, p 124
  - gaussian: adds a gaussian noise to the current value; the variance
    is a user-specified parameter
  - uniform: adds a uniform noise to the current value; the range is a
    user-specified parameter
- Two cross-over types are available (you can disable cross-over by
  using a cross\_rate equals to 0)
  - recombination:
    - a number k is randomly chosen
    - the first child is made of the [0, k[ values of the first
      parent and of the [k, n-1[ values of the second parent
    - the second child is made of the [0, k[ values of the second parent
      and of the [k, n-1] values of the first parent
  - SBX (simulated binary cross-over, cf Deb (2001), p 113). Warning:
    the current implementation is copied from Deb's code and is
    slightly different from the original paper.
- **Parameters:**
  - Any variant:
    - (mutation\_t and cross\_over\_t are defined in the namespace sferes::gen::evo\_float)
    - static const mutation\_t mutation\_type: polynomial, gaussian or uniform;
    - static const cross\_over\_t cross\_over\_type -> sbx or recombination;
    - static const float mutation\_rate -> the rate of mutation of one
      member of the vector
    - static const float cross\_rate -> the rate of cross-over (if no
      cross-over is applied, parents are copied to children)
  - polynomial mutation:
    - static const float eta\_m -> ηm is a parameter which controls
      the polynomial distribution. See Deb's paper or book.
  - gaussian mutation:
    - static const float sigma -> the standard deviation of the gaussian
  - uniform mutation:
    - static const float max -> maximum change of the current value
  - sbx:
    - static const float eta\_c -> see Deb's paper or book.
- **Typical typename:**

```
typedef sferes::gen::EvoFloat<10, Params1> gen_t;
```

- **Typical parameters:**

```
using namespace sferes::gen::evo_float;

struct Params1
{
  struct evo_float
  {
    static const float mutation_rate = 0.1f;
    static const float cross_rate = 0.1f;
    static const mutation_t mutation_type = polynomial;
    static const cross_over_t cross_over_type = sbx;
    static const float eta_m = 15.0f;
    static const float eta_c = 15.0f;
  };
};

struct Params2
{
  struct evo_float
  {
    static const float mutation_rate = 0.1f;
    static const float cross_rate = 0.1f;
    static const mutation_t mutation_type = gaussian;
    static const cross_over_t cross_over_type = recombination;
    static const float sigma = 0.3f;
  };
};


struct Params3
{
  struct evo_float
  {
    static const float mutation_rate = 0.1f;
    static const float cross_rate = 0.1f;
    static const mutation_t mutation_type = uniform;
    static const cross_over_t cross_over_type = sbx;
    static const float max = 0.3f;
    static const float eta_c = 15.0f;
  };
};
```

#### 2.5.3 Defining your own genotype

```
#include <sferes/stc/stc.hpp>
namespace sferes
{
  namespace gen
  {  
    SFERES_CLASS(MyGenotype)
    {
    public:
      // generates a random genotype
      void random()
      {
      // your code
      }
      // mutates an individual
      void mutate() 
      { 
      // your code to mutate (you have to handle your own mutation_rate)
      }
      // WARNING: the arguments of this method are of the same type
      // than your genotype
      void cross(const MyGenotype& o, MyGenotype& c1, MyGenotype& c2)
      {
      // if you don't know what to do, simply use:
      if (misc::flip_coin())
        {
          c1 = *this;
          c2 = o;
          }
        else
          {
            c2 = *this;
            c1 = o;
          }   
      }
    protected:
      // your data here (your genotype)
    };
  }
}
```

### 2.6 Phenotypes (Individuals)

Phenotypes are the expression of the genotypes: it is often useful to
develop a genotype into a structure which is easier to evaluate. For
instance, a neural network can be encoded with a compact encoding
(genotype, e.g. a modular encoding which can repeat the same module several
times), then developped into a standard neural network (phenotype).

Phenotypes are also the bridge between the fitness function and the
genotype. In sferes2, a phenotype is the composition of a fit\_t and
a gen\_t.

#### 2.6.1 Indiv

- **File:** sferes/phen/indiv.hpp
- **Description:** The abstract base class for phenotypes.
- **Main public methods:**

```
// returns the fitness
Fit& fit();
const Fit& fit() const;
// returns the genotype
Gen& gen()  { return _gen; }
const Gen& gen() const { return _gen; }
// develops the genotype, called by the evalutar before evaluating
// the individual
void develop();
// you can also override the following methods (although in most
// cases, you should override their equivalent in the genotype)
void mutate();
void random();
void cross(const boost::shared_ptr<Exact> i2, 
         boost::shared_ptr<Exact>& o1, 
         boost::shared_ptr<Exact>& o2);
// this method is not implemented in Indiv but it must be in all the
// concrete genotypes. It should describe this particular individual
// in the stream os (see the Parameter source code for an example)
void show(std::ostream& os) const
```

- **Defined types:**
  - Fit -> the fitness functor
  - Gen -> genotype

#### 2.6.2 Parameters

- **File:** sferes/gen/parameters.hpp
- **Unit test:** sferes/tests/phen/parameters.cpp
- **Description:** This phenotype transforms a list of parameters in
  [0, 1[ (typically a gen::Bitstring or a gen::EvoFloat) to a list of
  parameter in [min, max[. The genotype class must define a method:

```
cont std::vector<float>& data() const ();
```

- **Typical typename:**

```
typedef eval::Parameters<gen_t, fit_t, Params> phen_t;
```

#### 2.6.3 Defining your own phenotype

```
#include <sferes/phen/indiv.hpp>
namespace sferes
{
  namespace phen
  {
    SFERES_INDIV(MyPhenotype, Indiv)
    {
    public:
      // constructor
      MyPhenotype() { /* your code */ }
      // develop the genotype to whatever you want
      void develop(){ /* your code */ }
      // describe this individual
      void show(std::ostream& os) const { /* your code */ }
    protected:
      // your data
    };
  }
}
```

### 2.7 Evaluators

Evaluators are in charge of evalutating a vector of individuals
(i.e. running phen\_t::fit::eval() for each individual).

#### 2.7.1 Eval

- **File:** sferes/eval/eval.hpp
- A basic evaluator (no multiprocessing, no MPI, etc.), i.e. a basic
  loop.
- **Typical typename:**

```
typedef eval::Eval<Params> eval_t;
```

#### 2.7.2 Parallel

- **File:** sferes/eval/parallel.hpp
- **Description:** A SMP/multicore evaluator based on TBB
  (http://www.threadingbuildingblocks.org/). **Your fitness should be thread-safe!**
- **Typical typename:**

```
typedef eval::Parallel<Params> eval_t;
```

#### 2.7.3 Mpi

- **File:** sferes/eval/mpi.hpp
- **Description:** A MPI evaluator based on boost::mpi. It works on clusters (we use it
  on a JS22 cluster with IBM MPI).
- **Typical typename:**

```
typedef eval::Mpi<Params> eval_t;
```

#### 2.7.4 Defining your own evaluator

The only required method is eval. It should call phen\_t::develop()
then phen\_t::fit::eval() for each individual.

```
namespace sferes
{
  namespace eval
  {
    SFERES_EVAL(Eval)
    {
    public:
      template<typename Phen>
        void eval(std::vector<boost::shared_ptr<Phen> >& pop, size_t begin, size_t end)
      {
        for (size_t i = begin; i < end; ++i)
          {
            pop[i]->develop();
            pop[i]->fit().eval(*pop[i]);
          }
      }
    protected:
    };
  }
}
```

### 2.8 Modifiers

Modifiers are run once all the individuals have been evaluated but
before any sorting. They are designed to allow to modify the fitness
values to implement niching strategies, diversity preservation
mechanisms, etc.

The only predefined modifier available in sferes2 is
modif::Dummy, which does nothing.

#### 2.8.1 Dummy

- **File:** sferes/modif/dummy.hpp
- **Description:** basic modifier which does nothing.

### 2.9 Misc

The misc namespace contains useful small functions for:

- system access (e.g getpid())
- random number generation

## 3 Modules

### 3.1 NN

### 3.2 Fastsim

### 3.3 Python

{back to main page}

## 4 Writing modules

Author: Jean-Baptiste Mouret
<mouret@isir.fr>

Date: 2010-01-31 20:59:49 CET

HTML generated by org-mode 6.28e in emacs 23
