# Supplementary material for: To Cooperate or Not to Cooperate: Why Behavioural Mechanisms Matter
Source: PLoS Comput Biol. 2016 May 5;12(5):e1004886. doi: 10.1371/journal.pcbi.1004886 (PMC4858277; doi:10.1371/journal.pcbi.1004886)
Supplement: S1 Code — (GZ) [file pcbi.1004886.s005.gz › StagHuntExperiments/RoboticExperiments/doc/old/html/compilation.html]

xml version="1.0" encoding="iso-8859-1"?


Sferes2 – compilation


# Sferes2 – compilation

{back to main page}

## Table of Contents

- 1 Dependencies
- 2 Compilation 
  - 2.1 Simple compilation
  - 2.2 Configuration and details

## 1 Dependencies

We list here the Ubuntu package names:
We list here the Ubuntu package names:

- [required] libboost-dev >= 1.35 (including test, serialization, graph,
  programoptions, filesystem); see http://www.boost.org;
- [required] libboost-test-dev >= 1.35
- [required] libboost-filesystem-dev >= 1.35
- [required] libboost-program-options-dev >= 1.35
- [required] libboost-graph-parallel-dev >= 1.35
- [required] python >= 2.4;
- [required] g++ >= 4.2 (this should work with icc too);
- [optional but highly recommended] libtbb-dev; see
  http://www.threadingbuildingblocks.org/
- [optional but recommended] libeigen2-dev >= 2.06; see
  http://eigen.tuxfamily.org/
- [optional but recommended] python-simplejson;
- [optional] libboost-mpi-dev , if you need the optional MPI support (Warning: the
  Ubuntu packages of boost are not compiled with MPI support; you will
  have to compile boost yourself to use MPI).
- [optional] OpenMPI or another MPI implementation (tested with IBM
  MPI and OpenMPI)
- [optional] libgoogle-perftools-dev.

## 2 Compilation

### 2.1 Simple compilation

Assuming that everything has been properly installed you should be
able to compile sferes2 by typing (in the main sferes directory):

```
./waf configure
./waf build
```

You should then run the unit tests:

```
./waf check
```

If everything is green, sferes2 is working! If not, please send an
e-mail to the authors with as many details as
possible about your problem.

### 2.2 Configuration and details

Sferes2 employs the waf build system (http://code.google.com/p/waf/) instead of autoconf/automake and make. Waf employs files named
*wscript* to describe the compilation process in
python.

The main wscript of sferes2 accepts the following configuration options:

- –boost-includes=BOOSTINCLUDES : path to the boost directory where
  the includes are e.g. /usr/local/include/boost-1\_35
- –boost-libs=BOOSTLIBS : path to the directory where the boost libs
  are e.g. /usr/local/lib
- –no-mpi : disable mpi (MPI is automatically disabled if no MPI
  implementation is found)
- –mpi=MPI : path to MPI [e.g. /usr/lib/openmpi ]
- –apple : enable Apple (mac OSX) support
- –tbb=TBB : path to TBB if it's not installed in the default
  directory
- –64bits : compile for 64 bits architectures
- –ppc : compile for PowerPC (tested on IBM Power6)
- –rpath : set an optional rpath (useful in case of link failure)

To use these options, pass them to the waf script during the
configuration step. For example:

```
./waf configure --mpi /usr/lib/openmpi/
```

The configure options used during the last call of the "configure"
command are in the file build/configure.options.

Author: Jean-Baptiste Mouret
<mouret@isir.fr>

Date: 2010-01-31 20:59:49 CET

HTML generated by org-mode 6.28e in emacs 23
