# Supplementary material for: To Cooperate or Not to Cooperate: Why Behavioural Mechanisms Matter
Source: PLoS Comput Biol. 2016 May 5;12(5):e1004886. doi: 10.1371/journal.pcbi.1004886 (PMC4858277; doi:10.1371/journal.pcbi.1004886)
Supplement: S1 Code — (GZ) [file pcbi.1004886.s005.gz › StagHuntExperiments/RoboticExperiments/doc/old/html/sferes2.html]

xml version="1.0" encoding="iso-8859-1"?


Sferes2 – documentation


# Sferes2 – documentation

## Table of Contents

- 1 Introduction 
  - 1.1 Main features
  - 1.2 Authors
- 2 Where to go next?

## 1 Introduction

Sferes2 is a *framework* for evolutionary computation (EC) experiments
and especially for evolutionary robotics (ER). Its main goal is to
help researchers in EC and ER to efficiently try new ideas. Sferes2
has been inspired by sferes (http://sferes.lip6.fr), another (older
and no more maintained) framework for ER.

Sferes2 allows to design efficient programs on modern (multicore) computers: our
experiments typically require more than 24 hours of computation; in
this situation it can be profitable to trade some complexity of
implementation for some hours of computation. Nevertheless, we try to
keep the framework as simple as possible. More specifically, the
following choices were made in the initial design:

- use of modern c++ techniques (template-based programming)
  to employ object-oriented programming without the cost of virtual
  functions;
- use of Intel TBB to take full advantages of multicore and SMP systems;
- use of boost libraries when it's useful (shared\_ptr, serialization,
  filesystem, test,…);
- use of MPI to distribute the computational cost on clusters;
- a full set of unit tests;
- no configuration file: a fully optimized executable is built for
  each particular experiment.

This documentation assumes that the reader is reasonably familiar with
evolutionary algorithms. For references you can check the following
books about evolutionary computation:

- Deb, K. (2001). Multi-Objective Optimization Using Evolutionary
  Algorithms. *Wiley*.
- De Jong, K. A. (2002). Evolutionary Computation. *The MIT Press*.

### 1.1 Main features

Sferes2 currently includes the following EA and toolboxes (this is a
non-exhaustive list which contains only the most stable modules):

- EA
  - a rank-based single-objective EA
  - CMAES (Covariance Matrix Adaptation Evolution Strategie)
  - NSGA-II (multiobjective EA)
  - Epsilon-MOEA (multiobjective EA)
- Genotypes / Phenotypes:
  - Evolution of bit strings
  - Evolution of real parameters using different operators (SBX,
    gaussian, etc)
- Simulators
  - (optional) mobile robot (Khepera-like)
  - (optional) cartpole (single and double)
- Other
  - (optional) evolution of neural-networks

### 1.2 Authors

- Jean-Baptiste Mouret mouret@isir.fr: main author and
  maintainer
- Stephane Doncieux
- Many members of ISIR

## 2 Where to go next?

- Downloading sferes2
- How to compile sferes2 on your system
- A basic tutorial/example
- A reference manual / guide to the source code

Author: Jean-Baptiste Mouret
<mouret@isir.fr>

Date: 2010-01-31 21:01:48 CET

HTML generated by org-mode 6.28e in emacs 23
