# Supplementary figures and images for: To Cooperate or Not to Cooperate: Why Behavioural Mechanisms Matter
Source: PLoS Comput Biol. 2016 May 5;12(5):e1004886. doi: 10.1371/journal.pcbi.1004886 (PMC4858277; doi:10.1371/journal.pcbi.1004886)

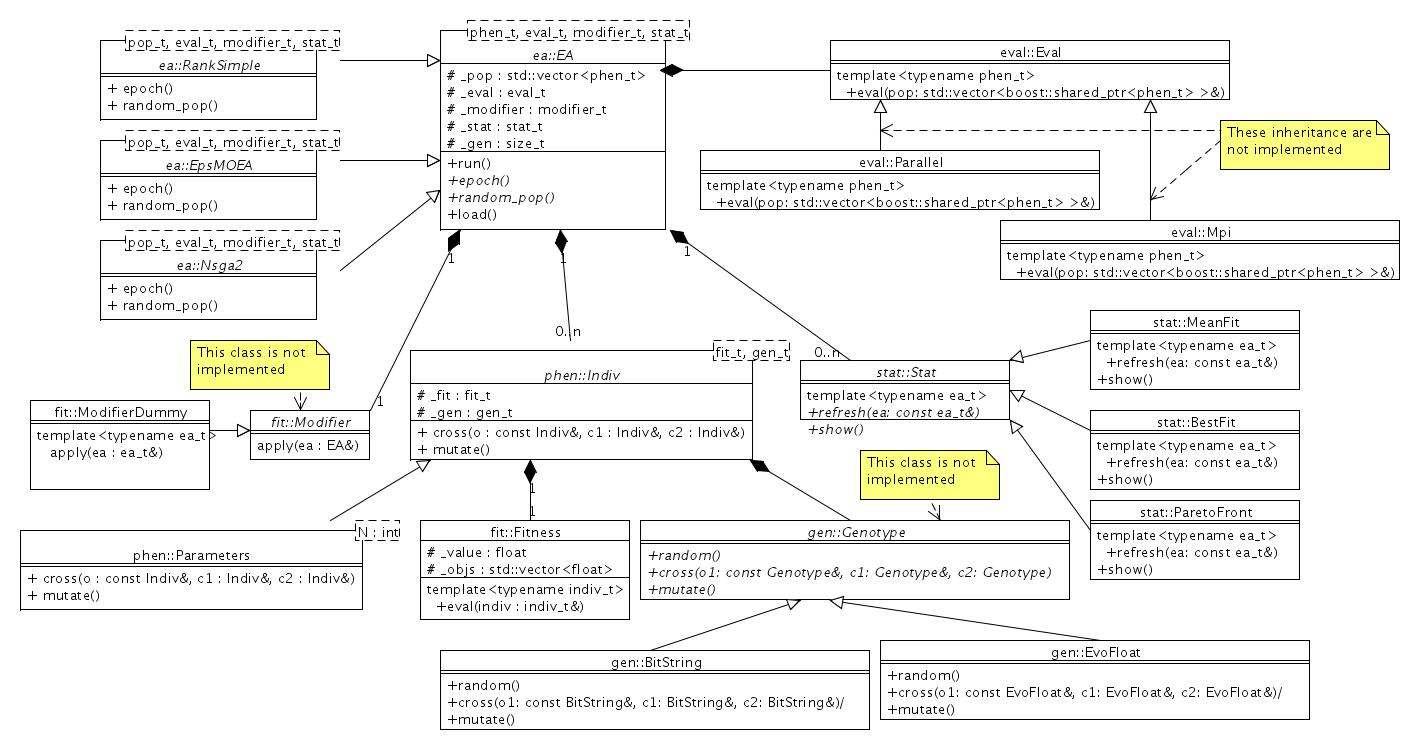

Supplement: S1 Code — (GZ) [file pcbi.1004886.s005.gz › StagHuntExperiments/RoboticExperiments/doc/old/img/sferes.jpg]

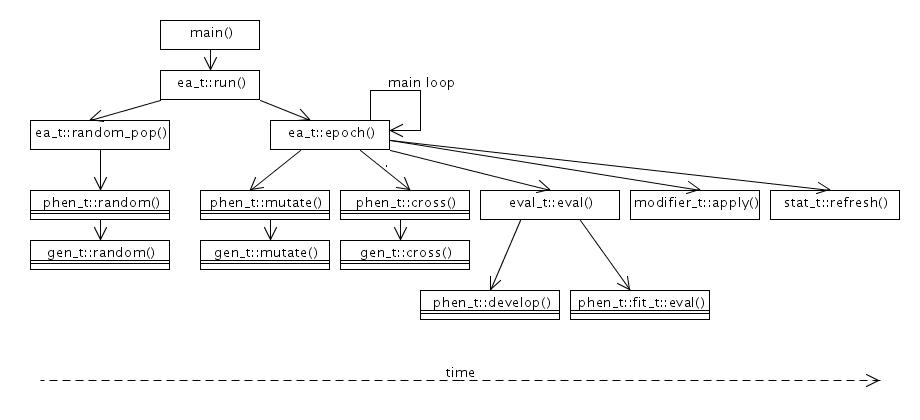

Supplement: S1 Code — (GZ) [file pcbi.1004886.s005.gz › StagHuntExperiments/RoboticExperiments/doc/old/img/call_graph.jpg]
