# Supplementary material for: To Cooperate or Not to Cooperate: Why Behavioural Mechanisms Matter
Source: PLoS Comput Biol. 2016 May 5;12(5):e1004886. doi: 10.1371/journal.pcbi.1004886 (PMC4858277; doi:10.1371/journal.pcbi.1004886)
Supplement: S1 Code — (GZ) [file pcbi.1004886.s005.gz › StagHuntExperiments/RoboticExperiments/doc/org/html/index.html.old]

Documentation 1.0


---

# Documentation 1.0

Copyright © 2011

CeCiLL

---

## Sferes 2

Introduction

Main features

Authors

Where to go next?

Specific sections for ISIR members

### Introduction

Sferes2 is a *framework* for evolutionary computation
(EC) experiments and especially for evolutionary robotics (ER). Its main
goal is to help researchers in EC and ER to efficiently try new ideas. Sferes2
has been inspired by sferes http://sferes.lip6.fr,
another (older and no more maintained) framework for ER.

Sferes2 allows to design efficient programs on modern (multicore) computers:
our experiments typically require more than 24 hours of computation; in this
situation it can be profitable to trade some complexity of implementation
for some hours of computation. Nevertheless, we try to keep the framework
as simple as possible. More specifically, the following choices were made
in the initial design:

- use of modern c++ techniques (template-based programming) to employ object-oriented
  programming without the cost of virtual functions;
- use of Intel TBB to take full advantages of multicore and SMP systems;
- use of boost libraries when it's useful (shared\_ptr, serialization, filesystem,
  test,...);
- use of MPI to distribute the computational cost on clusters;
- a full set of unit tests;
- no configuration file: a fully optimized executable is built for each
  particular experiment.

Appart from this documentation, information about Sferes 2 can be found in
Sferes\_v2: Evolvin'
in the Multi-Core World. If you use Sferes 2 in your work, please
cite the previous
publication.

The source code of many experiments using sferes is available on the evorob\_db website

This documentation assumes that the reader is reasonably familiar with evolutionary
algorithms. For references you can check the following books about evolutionary
computation:

- Deb, K. (2001). Multi-Objective Optimization Using Evolutionary Algorithms.
  *Wiley*.
- De Jong, K. A. (2002). Evolutionary Computation. *The MIT Press*.

### Main features

Sferes2 currently includes the following EA and toolboxes (this is a non-exhaustive
list which contains only the most stable modules):

- EA

  - a rank-based single-objective EA
  - CMAES (Covariance Matrix Adaptation Evolution Strategie)
  - NSGA-II (multiobjective EA)
  - Epsilon-MOEA (multiobjective EA)
- Genotypes / Phenotypes:

  - Evolution of bit strings
  - Evolution of real parameters using different operators (SBX, gaussian,
    etc)
- Simulators

  - (optional) mobile robot (Khepera-like)
  - (optional) cartpole (single and double)
- Other

  - (optional) evolution of neural-networks

### Authors

- Jean-Baptiste Mouret mouret@isir.fr:
  main author and maintainer
- Stephane Doncieux
- Paul Tonellitonelli@isir.upmc.fr
  : Documentation maintainer
- Many members of ISIR

### Where to go next?

Download Downloading sferes2

Compilation How to compile
sferes2 on your system

Tutorial/Example A tutorial/example
with explanation about the general structure of sferes2 experiment files,
the example file is generated automatically in the next tutorials.

Neurobiology Tutorial I
A tutorial to create an experiment using the neural network module of sferes2,
this enables you to create a neural network and evolve its weights and parameters.

Neurobiology Tutorial II
A tutorial to evolve the topology of a network in sferes2

Neurobiology Tutorial III
A tutorial to evolve a network of maps using the EvoNeuro module from sferes
II.

Reference A reference manual
/ guide to the source code

### Specific sections for ISIR members

Torque Introduction A reference
to launching experiments on the ISIR cluster

|  |  |
| --- | --- |
|  |  |

---
