# Supplementary material for: To Cooperate or Not to Cooperate: Why Behavioural Mechanisms Matter
Source: PLoS Comput Biol. 2016 May 5;12(5):e1004886. doi: 10.1371/journal.pcbi.1004886 (PMC4858277; doi:10.1371/journal.pcbi.1004886)
Supplement: S1 Code — (GZ) [file pcbi.1004886.s005.gz › StagHuntExperiments/RoboticExperiments/doc/old/html/tutorial.html]

xml version="1.0" encoding="iso-8859-1"?


Sferes2 – tutorial


# Sferes2 – tutorial

{back to main page}

This short example shows how to set up a basic experiment to optimize
parameters using a user-defined fitness.

## Table of Contents

- 1 Running a sferes2 executable
- 2 Loading a result file
- 3 Building your own experiment 
  - 3.1 Setting everything up 
    - 3.1.1 Create directories and files
    - 3.1.2 Compiling
    - 3.1.3 Running
  - 3.2 Customizing / writing the experiment 
    - 3.2.1 Main function
    - 3.2.2 Include part
    - 3.2.3 Params
    - 3.2.4 Fitness function

## 1 Running a sferes2 executable

Waf put all the compiled files in the "build" directory. Two variants
are produced: "default" and "debug". You should always run the debug
version first because the default version disables assert. Each
variant mirrors the architecture of the main sferes2 directory.

Sferes2 contains some basic examples in the "examples" directory. For
instance, let's check "ex\_ea.cpp", a basic single-objective
optimization of parameters. To launch the debug version, you should run:

```
build/debug/examples/ex_ea
```

A verbose mode is also available:

```
build/debug/examples/ex_ea -v all
```

And a more verbose one:

```
build/debug/examples/ex_ea -v all -v trace
```

For the optimized version:

```
build/default/examples/ex_ea
```

Running one of those commands should create a directory named using
the schema hostame\_pid-year-month-day\_hours:min:seconds. In this
directory there are:

- a file called "bestfit.dat", which contains, for each generation, the
  value of the best fitness;
- many files calles gen\_xxx where xxx is a number.

These files are XML files which store the best candidates
solutions for each generation (xxx is the generation number). For
instance:

```
<?xml version="1.0" encoding="UTF-8" standalone="yes" ?>
<!DOCTYPE boost_serialization>
<boost_serialization signature="serialization::archive" version="5">
  <x class_id="0" tracking_level="0" version="0">
    <_best class_id="1" tracking_level="0" version="1">
      <px class_id="2" tracking_level="1" version="0" object_id="_0">
        <_gen class_id="3" tracking_level="0" version="0">
          <_data>
            <count>10</count>
            <item_version>0</item_version>
            <item>0.50000423</item>
            <item>0.49999771</item>
            <item>0.49999756</item>
            <item>0.49999699</item>
            <item>0.50000197</item>
            <item>0.49999171</item>
            <item>0.5000003</item>
            <item>0.50000542</item>
            <item>0.50002229</item>
            <item>0.49999493</item>
          </_data>
        </_gen>
        <_fit class_id="5" tracking_level="0" version="0">
          <_value>-4.0586573e-14</_value>
          <_objs>
            <count>0</count>
            <item_version>0</item_version>
          </_objs>
        </_fit>
      </px>
    </_best>
  </x>
  <x class_id="6" tracking_level="0" version="0">
    <_mean>-5376.9888</_mean>
  </x>
</boost_serialization>
```

This XML file mirrors the statistics used in this experiment (more
about this in the next sections). At this point of this overview, you
can check:

- the \_data subtree which lists the values of the "optimal" parameters
  (the result of the experiment);
- the \_fit subtree which shows the fitness value of this "optimal" individual;
- the \_mean value which corresponds to the mean fitness in the population
  (another statistics);

## 2 Loading a result file

These XML files can be easily post-processed in your favorite language
if needed. However, the same executable which generated the result
files can also read them using the following syntax in the experience
directory :

```
../build/default/examples/ex_ea --load gen_1000 -o output_file -n number
```

- output\_file is a text file which, most of the time (depending on the
  genotype and phenotype used), describes the best individual (or the
  Pareto-optimal set in multiobjective optimization); in this
  example, it contains the value of the parameters for the best
  individual:

```
cat output_file
8.46386e-05 -4.58956e-05 -4.88758e-05 -6.02007e-05 3.93391e-05 -0.000165701 5.96046e-06 0.00010848 0.000445843 -0.000101328
```

- number is the number of the individual described in the file you want to load.

## 3 Building your own experiment

### 3.1 Setting everything up

#### 3.1.1 Create directories and files

- At the root of the sferes repository (the main directory), use waf
  to create a new experiment. Let's call it "test":

```
./waf --create test
```

This should have created a new directory exp/test, a waf file
exp/test/wscript and a basic file exp/test/test.cpp. You can now
edit/customize them.

#### 3.1.2 Compiling

- In the main sferes2 directory (not in the experiment's directory):

```
./waf --exp my_exp
```

If the experiment is called "test", the command line is:

```
./waf --exp test
```

#### 3.1.3 Running

```
cd exp/test
../../build/debug/exp/test/test
```

and for the optimized version:

```
../../build/default/exp/test/test
```

### 3.2 Customizing / writing the experiment

Let's start with exp/test/test.cpp, *from the end of the file*.

#### 3.2.1 Main function

At the end of the file, you should see a main() function. It defines
some types using typedef then run the evolutionary algorithm. The
types describe our experiment (what kind of genotype? what kind of fitness? what
kind of algorithm?). We will go back to the Params class in the following
section. Ignore it for now.

```
int main(int argc, char **argv)
{
  // Our fitness is the class FitTest (see above), that we call
  // fit_t. Params is the set of parameters (struct Params) defined in
  // this file.
  typedef FitTest<Params> fit_t;
  // We define the genotype. Here we choose EvoFloat (real
  // numbers). We evolve 10 real numbers, with the params defined in
  // Params (cf the beginning of this file)
  typedef gen::EvoFloat<10, Params> gen_t;
  // This genotype should be simply transformed into a vector of
  // parameters (phen::Parameters). The genotype could also have been
  // transformed into a shape, a neural network... The phenotype need
  // to know which fitness to use; we pass fit_t to it.
  typedef phen::Parameters<gen_t, fit_t, Params> phen_t;
  // The evaluator is in charge of distributing the evaluation of the
  // population. It can be simple eval::Eval (nothing special),
  // parallel (for multicore machines, eval::Parallel) or distributed
  // (for clusters, eval::Mpi).
  typedef eval::Eval<Params> eval_t;
  // Statistics gather data about the evolutionary process (mean
  // fitness, Pareto front, ...). Since they can also store the best
  // individuals, they are the container of our results. We can add as
  // many statistics as required thanks to the boost::fusion::vector.
  typedef boost::fusion::vector<stat::BestFit<phen_t, Params>, stat::MeanFit<Params> >  stat_t;
  // Modifiers are functors which are run once all individuals have
  // been evalutated. Their typical use is to add some evolutionary
  // pressures towards diversity (e.g. fitness sharing). Here we don't
  // use this feature. As a consequence we use a "dummy" modifier that
  // does nothing.
  typedef modif::Dummy<> modifier_t;
  // We can finally put everything together. RankSimple is the
  // evolutionary algorithm. It is parametrized by the phenotype, the
  // evaluator, the statistics list, the modifier and the general params.
  typedef ea::RankSimple<phen_t, eval_t, stat_t, modifier_t, Params> ea_t;
  // We now have a special class for our experiment: ea_t. The next
  // line instantiates an object of this class
  ea_t ea;
  // We can now process the command line options and run the
  // evolutionary algorithm (if a --load argument is passed, the file
  // is loaded; otherwise, the algorithm is launched).
  run_ea(argc, argv, ea);
  //
  return 0;
}
```

#### 3.2.2 Include part

Let's now go back to the top of the file. The file starts with the
usual include files, which obviously depends on which classes
(genotype, phenotype, ea, …) you selected in the main function:

```
#include <iostream>
#include <sferes/phen/parameters.hpp>
#include <sferes/gen/evo_float.hpp>
#include <sferes/ea/rank_simple.hpp>
#include <sferes/eval/eval.hpp>
#include <sferes/stat/best_fit.hpp>
#include <sferes/stat/mean_fit.hpp>
#include <sferes/modif/dummy.hpp>
#include <sferes/run.hpp>
```

#### 3.2.3 Params

Then, the Params structure defines the parameters of the
algorithm. This particular way of setting them allows the compiler to
propagate constants to the whole programm (i.e. it replaces the
parameters in the code by their values), allowing some
optimizations. This parameters will depend on the algorithms you chose
to use in your main function.

```
struct Params
{
  struct evo_float
  {
    // we choose the polynomial mutation type
    static const mutation_t mutation_type = polynomial;
    // we choose the polynomial cross-over type
    static const cross_over_t cross_over_type = sbx;
    // the mutation rate of the real-valued vector
    static const float mutation_rate = 0.1f;
    // a parameter of the polynomial mutation
    static const float eta_m = 15.0f;
    // a parameter of the polynomial cross-over
    static const float eta_c = 10.0f;
  };
  struct pop
  {
    // size of the population
    static const unsigned size = 200;
    // number of generations
    static const unsigned nb_gen = 2000;
    // how often should the result file be written (here, each 5
    // generation)
    static const int dump_period = 5;
    // how many individuals should be created during the random
    // generation process?
    static const int initial_aleat = 1;
    // used by RankSimple to select the pressure
    static const float coeff = 1.1f;
    // the number of individuals which are kept from one generation to
    // another (elitism)
    static const float keep_rate = 0.6f;    
  };
  struct parameters
  {
    // maximum value of the phenotype parameters
    static const float min = -10.0f;
    // minimum value
    static const float max = 10.0f;
  };
};
```

#### 3.2.4 Fitness function

Last, it's time to write the fitness function. It's a special class
with an "eval()" function which derives from fit::Fitness. It has to
fill this->\_value in single-objective optimization and
this->\_objs in multiobjective optimization. In this example, we want
to maximize -∑_i_ p_i_^4^, where p is the individual's phenotype.

```
SFERES_FITNESS(FitTest, sferes::fit::Fitness)
{
 public:
  // indiv will have the type defined in the main (phen_t)
  template<typename Indiv>
    void eval(const Indiv& ind) 
  {
    float v = 0;
    for (unsigned i = 0; i < ind.size(); ++i)
      {
        float p = ind.data(i);
        v += p * p * p * p;
      }
    this->_value = -v;
  }
};
```

{back to main page}

Author: Jean-Baptiste Mouret
<mouret@isir.fr>

Date: 2010-01-31 20:59:46 CET

HTML generated by org-mode 6.28e in emacs 23
